# Lower Striatal and Cortical Calretinin Interneuron Density Associated With Altered Social Behavior in *Cntnap2* Knockout Mice

**DOI:** 10.1002/aur.70286

**Published:** 2026-06-06

**Authors:** Krisztina Sáfár, Vivien Szendi, Paulina Hoppa, Yutong Wang, Fanni Á. Seres, Szilvia Bartók, Tyler Teadora, Lei Shi, Árpád Dobolyi, Gina Puska, István Adorján

**Affiliations:** ^1^ Department of Anatomy, Histology and Embryology, Faculty of Medicine Semmelweis University Budapest Hungary; ^2^ Laboratory of Molecular and Systems Neurobiology, Department of Physiology and Neurobiology Eötvös Loránd University Budapest Hungary; ^3^ JNU‐HKUST Joint Laboratory for Neuroscience and Innovative Drug Research College of Pharmacy, Jinan University Guangzhou Guangdong China; ^4^ Department of Zoology University of Veterinary Medicine Budapest Budapest Hungary

**Keywords:** autism spectrum disorder, calretinin, CNTNAP2, parvalbumin, social behavior, social novelty preference, striatum

## Abstract

Variants in the CNTNAP2 gene, encoding the cell adhesion molecule CASPR2, have been identified as genetic risk factors for autism spectrum disorder (ASD). However, the mechanisms through which CNTNAP2 dysfunction alters circuit function remain unknown. Interneurons, as key regulators of excitatory–inhibitory balance, represent a candidate source of vulnerability. In this study, we quantified calretinin‐positive (CR+) and parvalbumin‐positive (PV+) interneuron density in the caudoputamen (CP) and somatosensory cortex (SSC) of *Cntnap2* knockout (KO) and wild‐type (WT) mice and assessed their relationship with social behavior. *Cntnap2* KO mice exhibited significantly lower CR+ interneuron density in both brain regions, whereas no significant difference was observed in the PV+ density. *Cntnap2* KO females showed altered behavior in the social novelty preference test compared to WT females. *Cntnap2* KO animals also displayed elevated “moving away” responses, a social withdrawal phenotype. Correlation analyses revealed that within the KO group, higher striatal CR+ density was associated with a greater frequency and duration of “moving away.” CR+ density in the SSC did not correlate with this behavioral phenotype. The results suggest that striatal CR+ interneurons may modulate the persistence and intensity of social withdrawal behavior. In conclusion, our findings reveal region‐specific alterations in CR+ interneuron density in *Cntnap2* KO mice and uncover a previously unrecognized link between *Cntnap2* function, striatal interneuron organization and social withdrawal behavior. The results highlight CR+ interneurons as potential contributors to altered basal ganglia function in ASD and underscore the need for circuit‐level analyses of genetically defined risk models.

## Lay Summary

Mice missing the autism‐related *Cntnap2* gene showed changes in social behavior, including a tendency to avoid other mice and differences between males and females in social preference. The density of calretinin neurons was also lower in certain brain regions that play an important role in social behavior and movement control. We also found a relationship between withdrawal behavior and the calretinin interneuron density in the caudoputamen. The altered density of calretinin neurons may disturb brain circuits involved in social interaction, offering insight into how similar changes could affect social behavior in autism.

## 1 Introduction

Autism spectrum disorder (ASD) is a heterogeneous neurodevelopmental disorder defined by a diverse yet consistent set of core symptoms, including deficits in social communication and repetitive, stereotypic behavior (DSM‐5‐TR, American Psychiatric Association 2022). ASD affects an estimated 0.65% of the global population (Zeidan et al. 2022) and its etiology is thought to involve a complex interplay between genetic predisposition and environmental factors (Pugsley et al. 2022). In the past two decades, several genes have been connected to the development of ASD (Rylaarsdam and Guemez‐Gamboa 2019; Larsen et al. 2025), many of which have been shown to converge on neurodevelopmental processes (Paulsen et al. 2022). One of these genes is the contactin‐associated protein‐like 2 (*CNTNAP2*), which was first implicated in cortical dysplasia‐focal epilepsy (CDFE) (Strauss et al. 2006), a syndrome that shows high comorbidity with autism (Fujimoto et al. 2021).

*CNTNAP2/Cntnap2* encodes CASPR2/Caspr2, a neuronal cell adhesion molecule predominantly expressed in the nervous system, where it plays a crucial role in synapse formation and stabilization by organizing voltage‐gated potassium (K^+^) channels in the juxta‐paranodal region of myelinated axons (Poliak et al. 1999, 2001). *CNTNAP2/Cntnap2* expression begins during embryonic development and continues postnatally in both humans and mice, with prominent expression along the corticostriatal pathway in both species (Abrahams et al. 2007; Alarcón et al. 2008; Bakkaloglu et al. 2008; Vogt et al. 2017). Mutations of the gene can be associated with ASD‐related traits, such as deficits in language development and repetitive behavior (Alarcón et al. 2008; Arking et al. 2008; Bakkaloglu et al. 2008; Strauss et al. 2006). As in humans, the mutation or loss of *Cntnap2* can cause anatomical, physiological, and behavioral changes related to CDFE syndrome and ASD‐like features in mice (Peñagarikano et al. 2011). Absence of *Cntnap2* affects neuronal migration and leads to altered cellular density in the somatosensory cortex (SSC). Moreover, *Cntnap2* KO mice have been shown to spend significantly less time interacting with littermates and significantly more time with repetitive behavior phenomena such as self‐grooming and digging (Peñagarikano et al. 2011).

The imbalance of the excitatory/inhibitory (E/I) system could be a major contributor to the development of ASD and other neuropsychiatric disorders (Rubenstein and Merzenich 2003; Sohal and Rubenstein 2019). The parvalbumin (PV) hypothesis of autism states that dysfunction of PV+ interneurons disrupts inhibitory neural circuits, leading to E/I imbalance (Filice et al. 2020). This hypothesis is supported by the observation of lower density of PV+ cells (Ariza et al. 2018; Hashemi et al. 2016) and the downregulation of *PVALB* gene expression in patients with ASD (Parikshak et al. 2016; Schwede et al. 2018; Soghomonian et al. 2017). Similar patterns have been described in animal models of ASD (Deemyad et al. 2022; Lauber et al. 2016, 2018). E/I imbalance affects social novelty preference in *Cntnap2* KO mice, as rescuing E/I imbalance in the prefrontal cortex elevates the interaction time and social exploration of *Cntnap2* KO male mice compared to WT levels (Selimbeyoglu et al. 2017).

While PV+ and other interneuron subtypes have been studied extensively in ASD research, CR+ interneurons have received limited attention. The few human postmortem studies available report inconsistent findings: higher CR+ density in hippocampal CA1 (Lawrence et al. 2010), no change in the prefrontal cortex (Hashemi et al. 2016), and lower density in the caudate nucleus and anterior cingulate cortex (Adorjan et al. 2017). In the *Cntnap2* KO model, most studies have focused on PV+ or interneurons partially overlapping with CR+, with only one report noting lower density of CR+ neurons in the caudoputamen (CP) (Peñagarikano et al. 2011). Moreover, transcriptomic data from the neocortex revealed strong downregulation of several differentially expressed genes in vasoactive intestinal polypeptide+ (VIP) interneurons in ASD (Velmeshev et al. 2019)—a population that substantially overlaps with CR+ neurons (Hodge et al. 2019). However, the degree of overlap depends on the brain region, and can vary between cortical layers. Overall, in rodents it is estimated to be around 35%–95% (Guet‐McCreight et al. 2020; Posłuszny 2019; Xu et al. 2010). In our study, we focused on CR expressing interneurons as a broader phenotype. These findings suggest a potentially important but overlooked role for CR+ interneurons in ASD. Furthermore, the role of CR+ neurons in regulating social behavior in rodents is also not well‐established. To address the gaps in our knowledge regarding the role of CR+ interneurons in ASD, in this study, we compare the densities of CR+ and PV+ cells in the CP and the SSC between *Cntnap2* KO and wild‐type (WT) mice that were previously subjected to behavioral tests for correlative analysis. The CP was selected based on previous reports demonstrating altered CR+ interneuron density in this region (Peñagarikano et al. 2011; Adorjan et al. 2017), while the SSC was included due to its strong anatomical and functional connectivity with the CP (Hintiryan et al. 2016), enabling assessment of interneuron organization across interconnected corticostriatal circuits.

## 2 Methods

### 2.1 Animals

A total of 24 animals were used in the study. Sample size was determined based on previous studies in the *Cntnap2* KO mouse model while adhering to ethical guidelines for animal use and ensuring inclusion of both sexes; no priori power analysis was performed. Six male and six female adult (6 months of age) B6.129 (Cg)‐Cntnap2 < tm1Pele > /J mice carrying loss‐of‐function *Cntnap2* alleles obtained from The Jackson Laboratories (Poliak et al. 2003; JAX stock #017482) were used in the experiments. In addition, six male and six female age‐ and sex‐matched wild‐type controls were also used. Heterozygous mice (*Cntnap2* ^*+/−*^) were used for breeding to generate *Cntnap2* ^−/−^ (henceforward called *Cntnap2* KO) and *Cntnap2* ^*+/+*^ (henceforward called wild‐type (WT) mice). No visible shaking or abnormal immobility were seen in Cntnap2 KO animals during handling of the animals and on the recorded videos from the behavior tests. Experiments were conducted following the guidelines of the European Union Directive (EU Directive 2010/63/EU) and the Committee on the Care and Use of Laboratory Animals of the Council on Animal Care at Eotvos Lorand University of Budapest, Hungary (PE/EA/00403‐4/2023).

The genotype of the animals was determined by PCR using the following primers: mCNTNAP2‐gen‐Mut‐R (CGC TTC CTC GTG CTT TAC GGT AT), mCNTNAP2‐gen‐Comm‐F (CTG CCA GCC CAG AAC TGG), mCNTNAP2‐gen‐Wild‐R (ACA CCA GGG GCA AGA ATT G) according to the protocol suggested by The Jackson Laboratories for this line (JAX stock #017482).

Same‐sex mice were housed in pairs 2 months before starting the tests to adjust to the new environment in time under a 12–12 h light–dark cycle, with food and water available ad libitum. Pairs were made of one *Cntnap2* KO mouse and one WT mouse. Newly paired animals were monitored repeatedly every day to follow their adjustment period and to determine any harmful aggressive behavior.

### 2.2 Behavioral Tests

#### 2.2.1 Social Novelty Test

For social novelty test, a three‐chamber apparatus (60 × 40 × 25 cm) was used. The apparatus is divided into three chambers interconnected by doors ensuring free access to all chambers. Two wire enclosures are found in the side chambers, while the center chamber is empty. Social novelty preference is measured by placing an unknown and a known conspecific into the wire enclosures separately. In all cases, stimulus mice were wild‐type conspecifics of the same sex as the test animal. Partner mice previously paired with the test animals were used as known conspecifics, while unknown subjects were mice that test animals had never met beforehand. Test animals were habituated to the apparatus by placing them into the device, where they could freely move, for 10 min on the day before testing and for 5 min on test day before placing the stimulus mice into the cages. Both unknown and known mice were located in the small cages of the three chambers for 10 min on the previous day of test start. Animal behavior was monitored by camera for 10 min and postevaluated with SMART Video Tracking Software v3.0 (Panlab Harvard Apparatus, USA). TriWise method was used for automatic tracking of mice in the apparatus. Global activity, traveled distance, and time spent in each component were measured by the software.

#### 2.2.2 Direct Social Interaction Test

Direct social interaction was measured for 10 min in a neutral environment using an open field apparatus. The apparatus is 40 × 40 × 60 cm and is open on top. Animals were habituated to the apparatus separately on the day before testing. For the direct social interaction test, a *Cntnap2* KO mouse and a WT mouse were placed into the apparatus. Their behaviors were monitored by a video camera and postevaluated manually using the open‐sourced Solomon Coder software (made by András Péter). The following behavioral elements were rated: anogenital sniffing, body sniffing, mounting, body‐to‐body contact, chasing, approaching, moving away, passive social, and social proximity. If the evaluated animal was sniffing the anogenital region of the partner animal, the behavior was described as anogenital sniffing, while sniffing of any other body part was considered as body sniffing. Mounting was defined when the evaluated animal put both of its front paws on the partner and climbed on it. Body‐to‐body contact meant that the animals were standing next to each other with their bodies in contact. Chasing meant that the evaluated animal was following the partner animal while the partner was running away from it. In turn, approaching was defined as moving toward the partner animal while it did not move. Moving away describes two attached behaviors. First, moving away was used when the partner mice closely approached the test animals followed by the abrupt run away of the test animals. In addition, moving away includes the behavior sequence where partner mice interact with the test animals who then suddenly withdraw from the social stimuli. In both cases, partner mice might follow immediately or not the test animal. Passive social behavior was defined as active social engagement of the partner animal that was neither reciprocated nor followed by escaping behavior by the evaluated animal. Social proximity was described as close localizations of the animals without actual physical contact. The first appearance of each element (latency), the time spent with each element (duration), and the number of times each element appeared (number of elements) were evaluated for each animal.

### 2.3 Sample Preparation for Histology

Brain tissue from the same animals used in the behavioral tests was subjected to further histological analysis. Following the direct social interaction tests, animals were deeply anesthetized with ketamine‐xylazine solution, then transcardially perfused with 0.9% saline solution followed by 4% paraformaldehyde (PFA) in 0.1 M phosphate buffer (PB) solution. Brains were postfixed in 4% PFA 0.1 M PB solution. Brain hemispheres were separated; right hemispheres were postfixed with 10% formalin and embedded in paraffin. From the right hemispheres, 6 μm thick coronal sections were cut that were mounted on SuperFrost Plus slides, dried at RT and used for immunoperoxidase staining.

### 2.4 Immunohistochemistry

Sections were deparaffinized in xylene (2 × 5 min) and rehydrated through a graded ethanol series (100%, 96%, 90%, 70%; 1 min each). Endogenous peroxidase activity was blocked with 3% H_2_O_2_ in phosphate‐buffered saline (pH 7.4) for 20 min. Antigen retrieval was performed by heat‐induced epitope retrieval: CR sections were treated in citrate buffer (pH 6.0) in autoclave (121°C, 10 min), and PV sections in *tris*‐ethylenediaminetetraacetic acid buffer (pH 9.0) in microwave (3 min, maximum power). Slides were mounted in Sequenza System coverplates and racks (Thermo Fisher Scientific, 72110017, 73310017) and incubated at RT for 1 h with primary antibodies: rabbit anticalretinin (1:300, Chemicon, AB5054) or rabbit antiparvalbumin (1:500, Abcam, ab11427), diluted in *tris*‐buffered saline with 0.1% Triton X‐100 (pH 7.6). Detection was achieved using a horseradish peroxidase‐conjugated secondary antibody (EnVision Kit, Dako K‐5007, 1 h, RT) and visualized with 3,3′‐diaminobenzidine (DAB; 1:50 chromogen: substrate, 90 s). Slides were counterstained with hematoxylin (90 s), dehydrated through graded ethanol and xylene, and coverslipped with DePeX (Merck 06522).

### 2.5 Image Analysis

**FIGURE 1:**
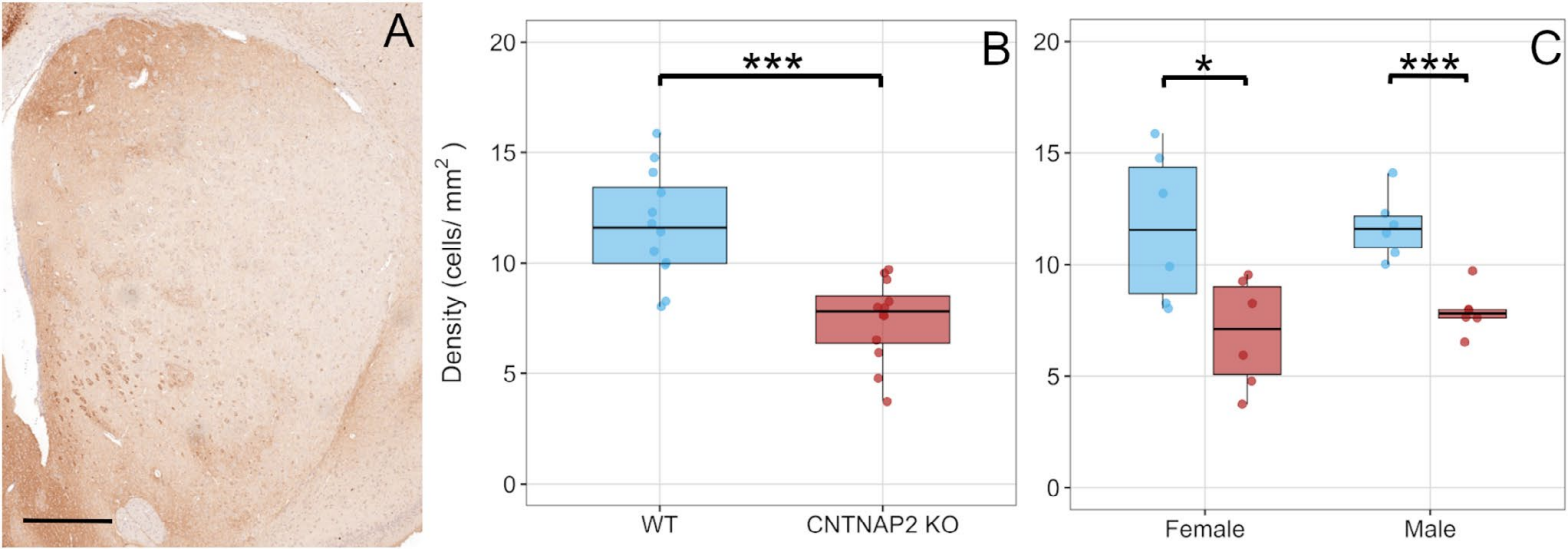
Significantly lower calretinin+ interneuron density in the caudoputamen of *Cntnap2* KO mice. (A) Representative image of the caudoputamen (CP) from a wild‐type (WT) animal. (B) Quantification of calretinin‐positive (CR+) interneuron density revealed significantly lower density in *Cntnap2* KO mice (red, *n* = 12) compared to WT mice (blue, *n* = 12). (C) Sex did not significantly affect CR+ interneuron density; the observed lower neuronal density remained significant in both females and males when analyzed separately. Cell density is presented as cells/mm^2^.

**FIGURE 2:**
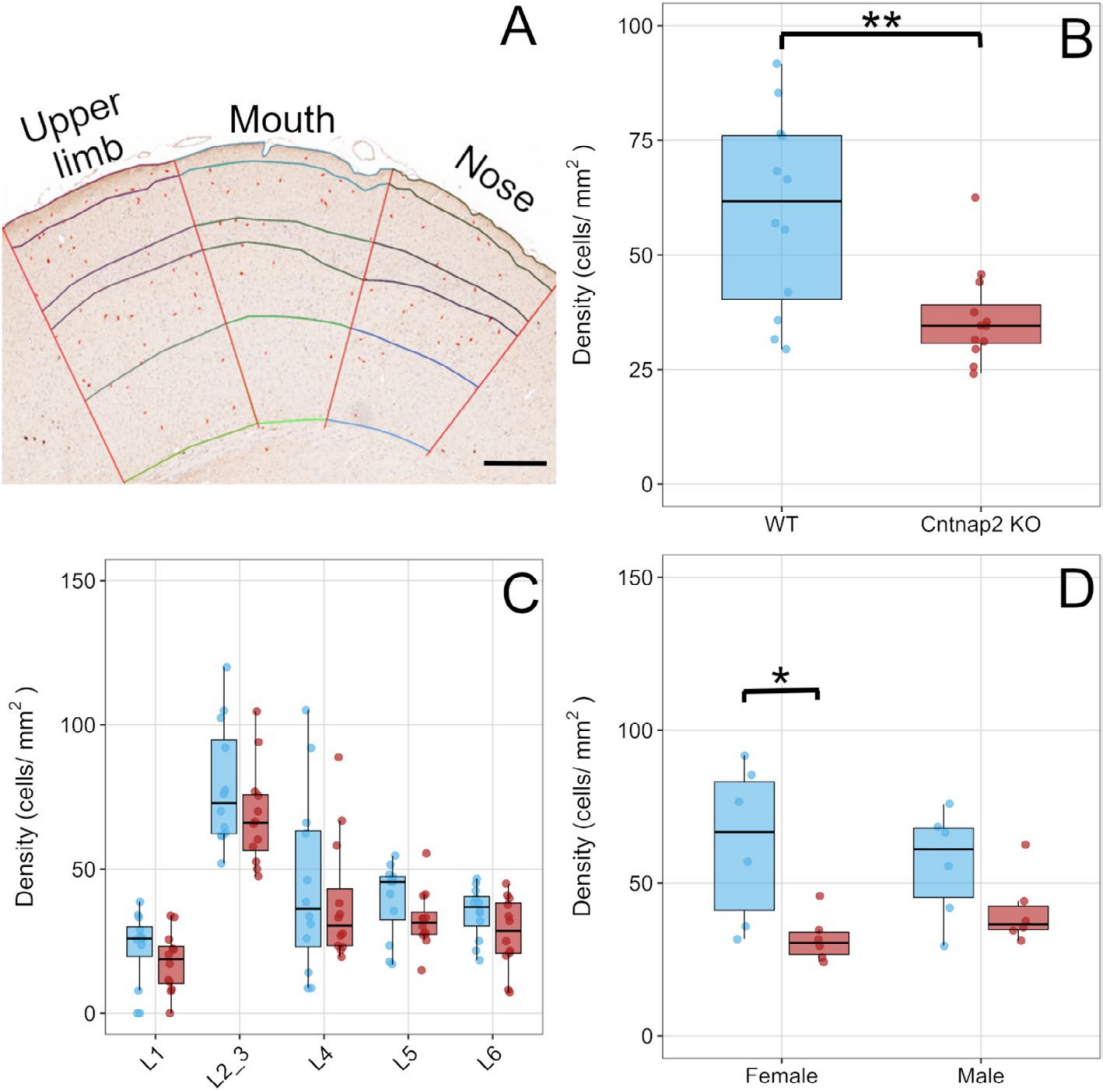
Lower calretinin+ interneuron density in the somatosensory cortex of *Cntnap2* KO mice. (A) Representative image of the somatosensory (SSC) with three subregions (upper limb, mouth, nose) and cortical layers delineated (L1–L6). (B) Quantification of calretinin‐positive (CR+) interneurons revealed significantly lower density only in the nose subregion in *Cntnap2* KO mice (red) compared to wild‐type (WT, blue). (C) Layerwise analysis of the nose subregion showed no significant difference in CR+ neuron density. (D) When analyzed by sex, CR+ interneuron density in the nose region of SSC was significantly lower in female *Cntnap2* KO mice compared to female WT mice, but it did not reach significance in males. Cell density is presented as cells/mm^2^.

Sections were digitized with a whole‐slide scanner (Pannoramic Flash Desk DX, 3DHistech). Image analysis was performed in QuPath (v0.4.4; Bankhead et al. 2017). Sections containing both regions of interest, CP (Figure 1A) and SSC (Figure 2A), were selected, with boundaries defined using the Allen Mouse Brain Atlas (Allen Institute for Brain Science 2004). The SSC was divided into three functional subregions: upper limb, mouth, and nose. Cortical layers were identified by cytoarchitectonic criteria with hematoxylin counterstaining. Immunopositive cell bodies were manually annotated at ×40 magnification if they showed a diameter > 4 μm, width > 2 μm, and a clear ovoid, ellipsoid, or fusiform morphology. Ambiguous profiles (e.g., fragmented or vascular‐associated cells) were excluded. Two sections were analyzed per animal. Cell density was calculated as cell number/area and reported as cells/mm^2^ ± SD. Cortical thickness was measured across SSC at 200 μm intervals.

### 2.6 Statistical Analysis

Statistical analysis was conducted with R software (v. 4.2.2.) with the packages lme4, lmerTest, and emmeans. Linear models (LM) were used to assess genotype (*Cntnap2* KO vs. WT) and sex effects on cell density in the CP. For the SSC, a linear mixed‐effects model (LMM) was used to test for diagnosis effects across cortical layers and subregions, with subject identity numbers included as a random intercept to account for repeated measures. The model included fixed effects for genotype, cortical layer, subregion, and sex. Nonsignificant interactions were removed based on likelihood ratio tests, and post hoc comparisons of estimated marginal means were conducted with correction for multiple comparisons, using Tukey's method, where appropriate. Model assumptions were assessed through Shapiro–Wilk test and visual inspection of normality plots.

Statistical analysis for the social novelty test was conducted using unpaired two‐tailed Student's *t*‐test in GraphPad Prism (v8.0.2). Behavioral data collected during the 10‐min social interaction tests were used to build a random forest (RF) classification model, implemented with 500 trees and a mtry parameter set to 3. Feature importance was evaluated using both mean decrease accuracy (MDA) and mean decrease gini (MDG) indices to identify the behavioral elements most predictive of ASD phenotype. MDA describes the reduction in model accuracy that occurs because of permuting the values in each predictor variable. The MDG is used to demonstrate the contribution of the variables to the reduction of class impurities across all trees (Fife and D'Onofrio 2023). The variables that demonstrated higher values than the calculated mean MDA and MDG indices were selected as the most informative variables for feature classification. RF classification models were implemented in RStudio (version 2025.09.0 Build 387) using the randomForest package. Subsequently, the top‐ranked behavioral features were analyzed individually using linear regression models to test group differences. In each model, the genotype (*Cntnap2* ^*−/−*^ vs. *Cntnap2* ^*+/+*^), sex, and their interaction were included as predictors. Statistical significance was set at *p* < 0.05 for all statistical analyses.

## 3 Results

### 3.1 Lower CR+ Interneuron Density in the Caudoputamen of Cntnap2 KO Mice

A total of 1954 CR+ interneurons were quantified in the CP across 12 *Cntnap2* KO and 12 WT mice. A significantly lower CR+ cell density was observed in *Cntnap2* KO animals, representing a 36% difference (WT: 11.69 ± 2.48, KO: 7.42 ± 1.85) compared to WT mice (Figure 1B; *p* = 0.0001, LM). Sex had no significant effect on cell density (*p* = 0.6075). Accordingly, when analyzed separately (Figure 1C), a 32% lower density was observed in males (WT: 11.70 + 1.44, KO: 7.91 + 1.03; *p* = 0.0004, LM) and a 40% lower density in females (WT: 11.68 ± 3.39, KO: 6.92 ± 2.44; *p* = 0.0192, LM).

### 3.2 CR+ Interneuron Density Is Lower in the Nose Subregion of the SSC

We next assessed CR+ interneuron density across subregions of the SSC (nose, mouth, and upper limb areas). Based on the analysis of 4597 cells, a statistically significant difference was found in the nose subregion, where *Cntnap2* KO mice exhibited a 39% lower density of CR+ cells compared to WT animals (Figure 2B; WT: 59.62 ± 14.37, KO: 36.35 ± 10.47; *p* = 0.0032, LM). To determine whether the observed difference was layer‐specific, we conducted a layerwise analysis of CR+ cell densities. No significant difference was found, suggesting that the lower density reflects a nonlayer‐specific, spatially consistent alteration (Figure 2C; Table S1). No significant differences were observed in the other subregions or the overall density in the whole SSC (Figure S1 and Table S1).

LM revealed no significant main effect of sex on cell density (Table S2). However, sex‐stratified analysis showed that the genotype effect was significant only in females (WT: 62.97 ± 23.52, KO: 31.84 ± 7.82; *p* = 0.0171), whereas males showed a similar but nonsignificant difference (WT: 56.26 ± 17.68, KO: 40.85 ± 11.46; *p* = 0.1033) (Figure 2D). These results suggest that the apparent sex‐specific difference may reflect individual variability rather than a robust biological effect.

### 3.3 PV+ Interneuron Density and Brain Macrostructure Remains Unaffected

Across the analyzed brain regions, the density of PV+ interneurons did not differ significantly between *Cntnap2* KO and WT animals (Figures S2 and S3 and Table S3). Sex had no significant effect on PV+ cell density (Table S4). To determine whether macrocephaly is present in the *Cntnap2* KO mouse model, we measured the area of the CP and the cortical thickness of the SSC. No significant differences were found between genotypes in any of the examined regions (Figure S4).

### 3.4 Cntnap2 KO Mice Showed Altered Preference Toward Unknown Conspecific

**FIGURE 3:**
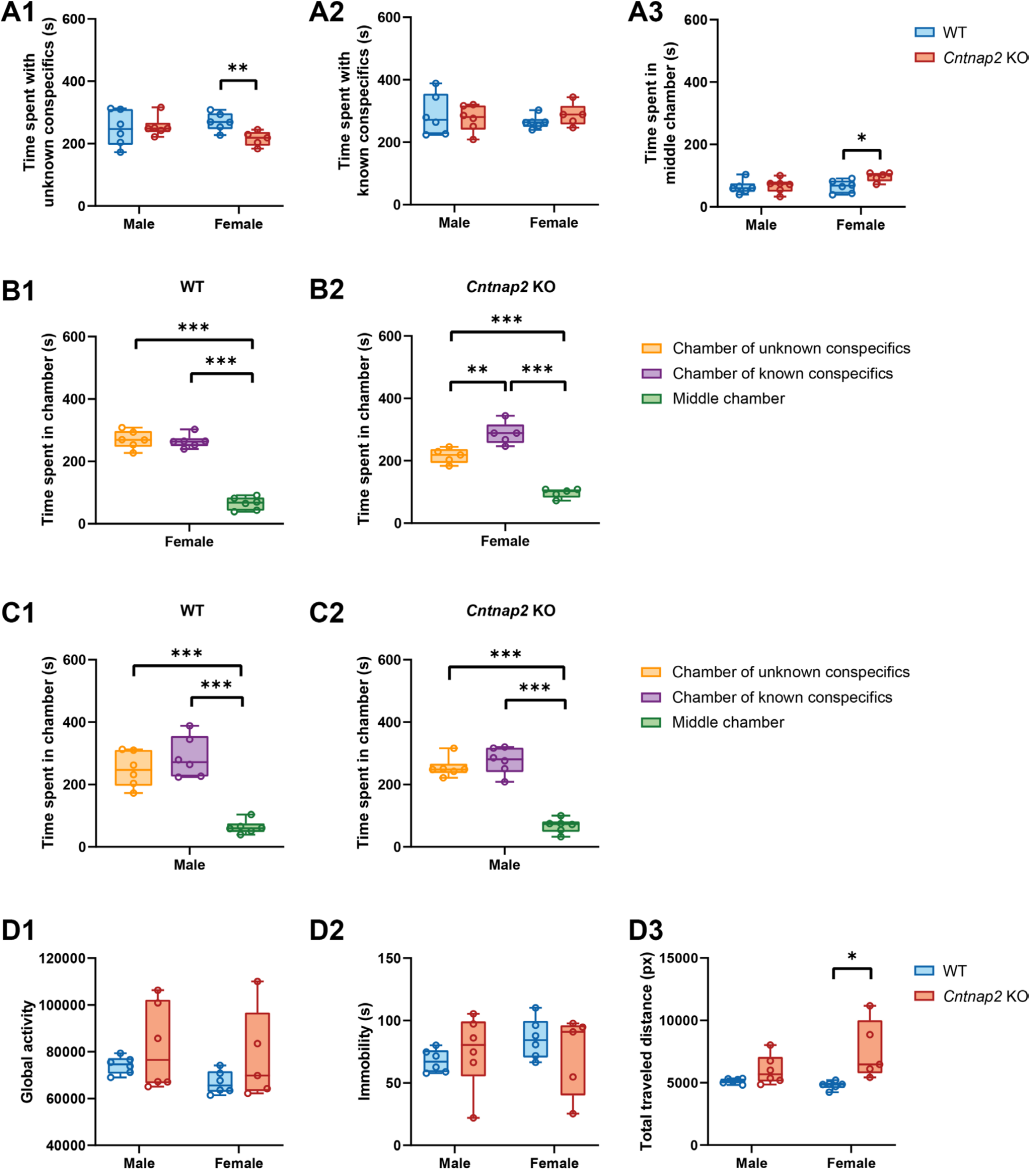
Significantly altered social novelty preference in *Cntnap2* KO females. (A1–A3) Summarization of the time spent in the different chambers by *Cntnap2* KO and wild‐type (WT) females and males (*N* = 6 in each group). *Cntnap2* KO females spent significantly less time in the chamber containing the unknown conspecifics compared to WT (A1, *p* = 0.0083), while no difference was seen in the time spent with known conspecifics (A2, *p* = 0.2287). Compared to WT females, *Cntnap2* KO females spent more time in the empty middle chamber (A3, *p* = 0.0191). In case of males, no significant changes were seen between *Cntnap2* KO and WT mice (A1–A3). (B1 and B2) Summary graphs of the comparison of time spent in the different chambers in case of females. B2 shows that *Cntnap2* KO females spent significantly more time with the known conspecifics compared to the unknown ones (*p* = 0.0063). This difference cannot be seen in the WT female group (B1, *p* = 0.7091). However, both groups spent significantly more time with either conspecifics compared to the empty middle chamber (*p* < 0.0001 every time). (C1 and C2) Summary graphs regarding males. *Cntnap2* KO males show no difference in time spent with the known or unknown conspecifics (C2, *p* = 0.3368). Similarly to the females, both male experimental groups spent significantly more time in the chamber of either conspecifics compared to the empty middle chamber (*p* < 0.0001 every time). (D1–D3) Graphs are showing activity‐related data of male and female mice comparing Cntnap2 KO and wild‐type groups. Global activity (D1) and immobility duration (D2) show no significant difference comparing Cntnap2 KO and wild‐type mice in either males or females. However, total traveled distance (D3) is significantly higher in Cntnap2 KO female mice than in wild‐type ones (*p* = 0.0176). Box plots display whiskers extending to the minimum and maximum observed values, with the box representing the interquartile range and the line inside the box indicating the median.

Social novelty preference testing in the three chamber apparatus revealed alteration in the behavior of female *Cntnap2* KO mice, as these mice spent significantly less time in the chamber containing the unknown conspecifics compared to the WT female group (Figure 3A1, *p* = 0.0083). Furthermore, *Cntnap2* KO females stayed significantly more time in the empty middle chamber than WT (Figure 3A3, *p* = 0.0191). No significant differences were seen regarding the time spent in the chamber of the known conspecific among the females (Figure 3A2). Comparing the time spent in each chamber by the *Cntnap2* KO females, we found that these animals showed preference toward the known conspecifics as they spent significantly more time with them compared to the unknown conspecifics (Figure 3B1, *p* = 0.0063). This difference was not present in the WT group (Figure 3B2). Both groups spent more time with either known or unknown conspecifics compared to the time spent in the empty middle chamber (Figure 3B1–B2, *p* < 0.0001 every time).

Neither *Cntnap2* KO males nor their WT showed altered behavior in the social novelty test. There was no significant difference in the time spent in the middle chamber (Figure 3A3) or the chamber containing the known (Figure 3A2) or the unknown (Figure 3A1) conspecifics compared to the WT. Furthermore, no significant changes were observed in the time spent with the unknown conspecific when compared to time spent with the known one in either male group (Figure 3C1–C2). Both groups spent more time with either conspecific compared to the time spent in the empty middle chamber (Figure 3C1–C2, *p* < 0.0001 every time). Additional statistical data can be found in Tables S5 and S6.

There was no difference in global activity and time spent with immobility across the examined groups (Figure 3D1–D2), while Cntnap2 KO female mice moved more than their wild‐type conspecifics according to the total traveled distance data (*p* = 0.0176; Figure 3D3) (Table S7).

### 3.5 *Cntnap2* KO Mice Exhibited Social Withdrawal

**FIGURE 4:**
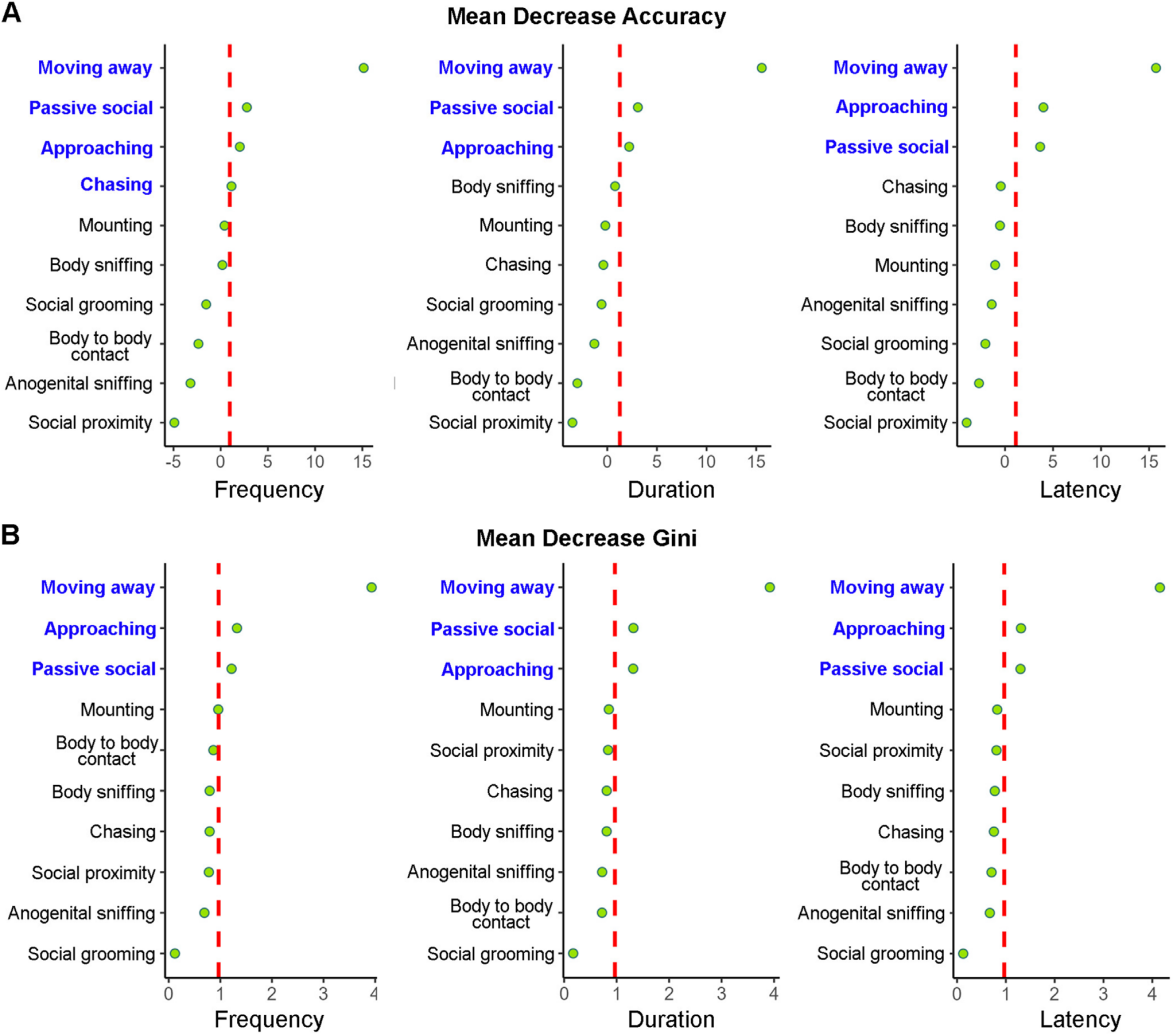
Behavioral feature importance based on random forest classification. The random forest classifier model was implemented with 500 trees and an mtry parameter set to 3. The points represent the mean decrease accuracy (MDA) (A) and mean decrease gini (MDG) (B) values of the frequency, duration, and latency of the behavioral elements, indicative of the importance of each variable. (A) The points represent the MDA values reflecting the impact of each feature on model accuracy. (B) The points represent the MDG values reflecting each feature's contribution to classification performance. Discontinuous vertical lines represent the mean value of the importance of each variable. Behavioral elements with MDA and MDG values above the mean were identified as the most important features distinguishing ASD and control groups.

To investigate whether *Cntnap2* KO phenotype is associated with altered social behavior, we conducted direct social interaction tests with *Cntnap2* KO and WT mice in the open field apparatus. To identify the behavioral elements most predictive of ASD phenotype, RF models were built separately for the three behavioral parameters (duration, frequency, and latency). RF provides robust estimates of variable importance in datasets with multiple predictors and captures nonlinear interactions among them (Breiman 2001). Mean decrease in accuracy (MDA) and mean decrease in Gini (MDG) values, which indicate the relative importance of the measured behavioral variables, were calculated for feature selection in the classification models. MDA quantifies the reduction in model accuracy resulting from permuting the values of each predictor variable. The MDG is used to demonstrate the contribution of the variables to the reduction of class impurities across all trees (Fife and D'Onofrio 2023). Variables with values exceeding the calculated mean of the MDA and MDG indices were selected as the most important variables for feature classification. Moving away, approaching, and passive social behavior were consistently ranked among the top predictors, based on both MDA (Figure 4A) and MDG (Figure 4B) indices.

**FIGURE 5:**
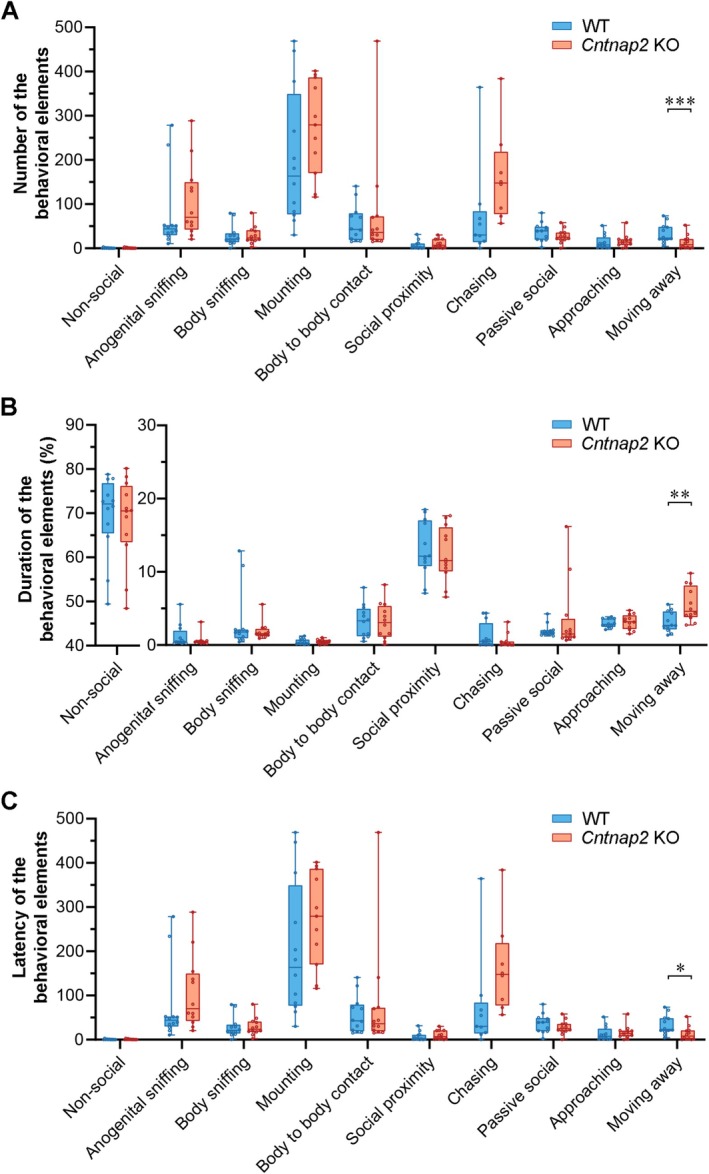
Behavioral change in social interactions of *Cntnap2* KO mice. Direct social interaction tests were conducted with Cntnap2 KO (*n* = 12) mice and WT (*n* = 12) mice. The frequency (number of behavioral elements per 10 min) (A), duration (B), and latency (C) of various social behavioral elements were measured. Latency values for animals that did not exhibit chasing behavior are not shown in the figure (4 out of 12 *Cntnap2* KO mice; 3 out of wild‐type mice). Box plots display whiskers extending to the minimum and maximum observed values, with the box representing the interquartile range and the line inside the box indicating the median. Linear regression models were used.

These behaviors were therefore selected and introduced into linear regression analyses to reveal behavioral differences between *Cntnap2* KO and WT mice. The experimental group had a significant effect on moving away behavior. *Cntnap2* KO mice exhibited a significantly higher frequency (KO: 39.83 ± 4.07 (SD); WT: 22.41 ± 2.88 (SD), *p* = 0.0003) (Figure 5A) and duration (KO: 33.33 ± 3.36 (SD); WT: 19.07 ± 4.76 (SD), *p* = 0.0066) (Figure 5B) of moving away behavior compared to WT mice. Consistent with this, the latency of moving away was significantly lower (*p* = 0.0191) in the *Cntnap2* KO group (13.48 ± 5.55 (SD)) compared to WT mice (33.32 ± 7.84 (SD)) (Figure 5C). Although a significant main effect of group was revealed, neither sex nor the Group × Sex interaction had a significant impact on the moving away behavior. Moreover, neither passive social behavior nor approaching showed significant differences between *Cntnap2* KO and WT mice. Additional statistical data are provided in Table S8. On the video recordings, no visible aggressive behavior was identified during male–male or female–female interactions.

### 3.6 Altered Calretinin Cell Density Is Associated With Altered Social Behavior in *Cntnap2* KO Mice

**FIGURE 6:**
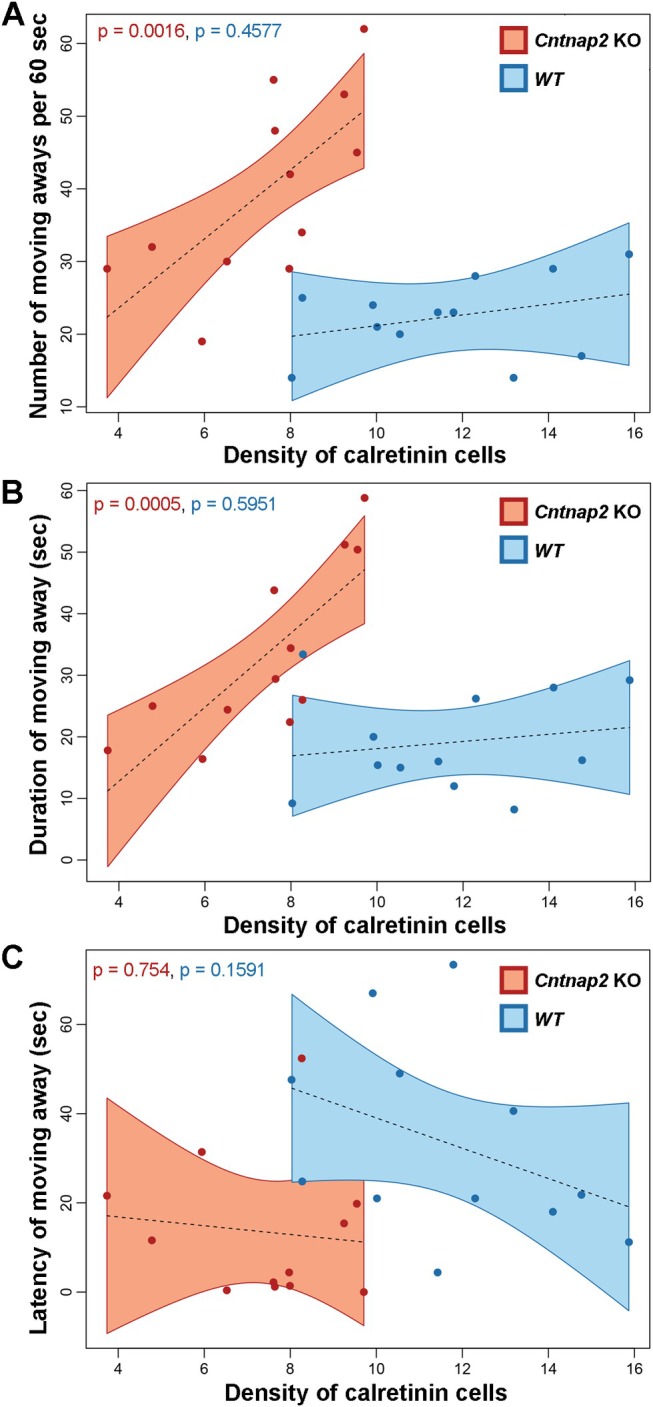
Correlation between calretinin‐immunopositive (CR+) cell density in the caudoputamen and moving away behavioral response of *Cntnap2* KO mice. The graphs show the frequency (A, number of behavioral elements per 10 min), the duration (B), and the latency of moving away behavior (C) in relation to CR+ cell densities in each examined group (*Cntnap2* KO (*n* = 12) and WT (*n* = 12) mice). Red dots represent individual values. Red and green lines indicate the estimated marginal means predicted by the linear mixed model. The colored areas represent estimated marginal means with 95% confidence intervals. The model confirmed that higher CR+ cell density is associated with higher frequency (*p* = 0.0016) and duration (*p* = 0.0005) of moving away behavior in Cntnap2 KO but not in WT (*p* = 0.458 and *p* = 0.595, respectively) mice.

Given the relevance of the “moving away” behavioral element to the *Cntnap2* KO related behavioral phenotype, an additional statistical analysis was conducted to reveal whether the altered CR cell density in the CP is correlated with the behavioral moving away response (Figure 6). There was a significant interaction between the examined groups and CR+ cell density in the CP, indicating that the relationship between CR+ cell density and the moving away behavior differed between *Cntnap2* KO and WT mice. The interaction was demonstrated in terms of both the frequency (*p* = 0.023) and the duration (*p* = 0.007) of the behavior, but not its latency. CR+ cell density exhibited a positive correlation with the frequency (*p* = 0.0016) and duration (*p* = 0.0005) of moving away behavior in the *Cntnap2* KO group, but not in the WT group (*p* = 0.458 for frequency, *p* = 0.595 for duration). Additional statistical data are provided in Table S9.

The altered CR density observed in the nose region of the SSC did not correlate with the moving away behavior in either group. Statistical data are provided in Table S10.

## 4 Discussion

### 4.1 Altered CR+ Interneuron Density in *Cntnap2* KO Mice

In this study, we analyzed CR+ and PV+ interneuron density in 12 *Cntnap2* KO and 12 WT mice, with equal numbers of males and females in each group. *Cntnap2* KO mice showed significantly lower CR+ cell density in the CP and the nose subregion of the SSC. Only one previous study has quantified CR+ interneurons in this model. Peñagarikano et al. (2011) reported fewer CR+ neurons in the SSC; however, they did not differentiate between subregions. Furthermore, although CP was included in their analysis of PV+ and neuropeptide Y interneurons, no data was presented regarding CR+ neurons in this region. Notably, Adorjan et al. (2017) also reported significantly lower density of CR+ interneurons in the caudate nucleus of individuals diagnosed with ASD, suggesting that reduced CR^+^ interneurons may represent a convergent neurobiological feature across species and models.

The lower CR+ interneuron density may have multiple underlying causes. One possibility is preserved neuronal presence with reduced CR expression, driven by disruptions during the transcription and/or translation of *Calb2/*CR. Alternatively, it may reflect genuine cell loss due to developmental dysfunction, altered migration, or selective degeneration. Both interneuron migration abnormalities (Peñagarikano et al. 2011) and PV downregulation without the loss of *Pvalb* mRNA (Lauber et al. 2018) have been described in *Cntnap2* KO mice, indicating that interneuron development and function are affected on multiple levels. Clarifying the underlying mechanism will require multiscale analyses; for example, multiplex RNAscope could simultaneously assess *Calb2* mRNA and CR protein levels to distinguish between cell loss and altered protein expression. In either case, the lack of CR could severely impact both the development and function of the inhibitory network.

As a hexa‐EF‐hand Ca^2+^‐binding protein, CR mainly acts as a calcium (Ca^2+^) buffer and sensor (Schwaller 2014). Loss of CR impairs several neuronal properties, including dendritic arborization (Su et al. 2021), intrinsic excitability (Cheron et al. 2000; Gall et al. 2003), synaptic plasticity (Schurmans et al. 1997) as well as proliferation and migration of progenitor cells (Todkar et al. 2012), underscoring its critical role in neuronal development and circuit function. As CR+ interneurons target both pyramidal cells and other inhibitory neurons (Saffari et al. 2019), their dysfunction may simultaneously alter excitatory and inhibitory compartments of the neuronal circuits. *Cntnap2* likely contributes to these processes from early development, since its expression was observed in the ganglionic eminences (Abrahams et al. 2007; Bakkaloglu et al. 2008). Although Cntnap2 expression has been reported in specific interneuron subtypes (e.g., PV+ and VIP+), its expression in CR+ interneurons is not well established, suggesting that observed CR+ alterations may arise indirectly. In *CNTNAP2* KO organoids, increased progenitor proliferation and organoid volume (de Jong et al. 2021), shortened cell cycles, enhanced surface folding, and altered expression of interneuron‐related transcription factors (Chalkiadaki et al. 2025) were described. Together, these findings suggest that *CNTNAP2/Cntnap2* dysfunction disrupts interneuron development more broadly, which may secondarily affect interneuron populations, including CR+ neurons, further compromising the developing inhibitory network. Such disturbances are likely to impair integration of somatosensory information within the corticostriatal network, where the SSC and CP form tightly coupled circuits critical for sensorimotor processing and social behavior.

We observed no significant difference in PV+ interneuron density between genotypes in either region, suggesting a selective vulnerability of CR+ interneurons in adult *Cntnap2* KO mice. Some previous studies observed no significant difference in the density of PV+ neurons in the striatum (Briones et al. 2022; Cording et al. 2025) and in the SSC (Gandhi et al. 2023). However, others reported lower PV+ neuron number or density in the CP (Lauber et al. 2018; Peñagarikano et al. 2011) and in the SSC (Deemyad et al. 2022; Peñagarikano et al. 2011; Vogt et al. 2017). Discrepancies across studies may arise from methodological differences detailed below.

### 4.2 Age and Sex as Key Variables in ASD Research

Previous *Cntnap2* KO studies focused on juvenile or adolescent mice (< P60; Arellano et al. 2024), thereby providing insights into early postnatal neurodevelopment but overlooking adulthood. However, adulthood represents a distinct developmental phase during which neuronal circuits and behavioral phenotypes may persist and stabilize. Ahn et al. (2017) found increased CR+ IN number in the SSC by 98% from postnatal month 1 (PM1) to PM6, which then declined by 29% at PM24, suggesting dynamic, age‐dependent fluctuations. Importantly, our analysis at PM6 was conducted at the developmental peak of CR+ numbers, thereby capturing these interneurons at their maximal abundance and allowing us to assess their long‐term contribution to corticostriatal circuitry. Sex is another key factor, yet, with few exceptions (Deemyad et al. 2022; Gandhi et al. 2023), most studies only analyzed males or did not specify sex. Given the rising number of female diagnoses and emerging evidence of sex‐specific phenotypes in ASD (Gellert et al. 2025; McCrossin 2022; Wood‐Downie et al. 2021), including females in experimental designs is essential for capturing the full spectrum of neurobiological alterations. Sample size is also an important source of variability. Earlier studies used 3–6 mice/genotype, whereas our study included 12 mice/genotype, the largest histological cohort for CR+ and PV+ interneurons in this model. Interestingly, our results showed no significant interaction between sex and genotype in the nose subregion of the SSC, however sex‐stratified analysis revealed a nonsignificant lower CR+ interneuron density in males. Sex‐stratified results should be interpreted with caution due to limited subgroup sample size, as the male subgroup showed reduced statistical power and wide confidence intervals. Therefore, in future studies larger, purpose‐designed cohorts will be required to reliably assess potential sex‐specific effects.

### 4.3 No Macrostructural Changes in *Cntnap2* KO Mice

Increased brain volume has been reported in some ASD cases and in CDFE syndrome (Sacco et al. 2015). To examine whether such a phenotype is present in the *Cntnap2* KO model, we measured CP area and cortical thickness of SSC but found no significant differences between KO and WT mice. Lauber et al. (2018) also observed no volumetric changes in SSC of *Cntnap2* KO animals. By contrast, cortical overgrowth has been described in humans with *CNTNAP2* deficiency and in patient‐derived organoids (de Jong et al. 2021). As noted by the authors, these findings suggest that certain phenotypes may be human‐specific and cannot be fully modeled in mice due to evolutionary differences in cortical development.

### 4.4 Behavioral Correlates of CR ^+^ Interneuron Alterations

The cellular changes found in the *Cntnap2* KO mice raise the question whether these alterations correlate to any behavioral abnormalities. Until now, various studies have examined ASD‐like repetitive behavioral phenotypes in the *Cntnap2* KO mice but mostly in males and juvenile or adolescent ones (Cording et al. 2025; Peñagarikano et al. 2011; Thabault et al. 2022), although Pengarikano et al. previously described no sexual dimorphism in the behavior of female mice (Peñagarikano et al. 2015). In addition, social deficits, which are also a behavioral phenotype of ASD, have been examined in *Cntnap2* KO mice. Sociability has been extensively examined in young 4–8 week old mice (Peñagarikano et al. 2011, 2015), and also in older 12–14 week old cohort (Mohapatra et al. 2025), and in these researches no sexual dimorphism was found thus results of male and female mice were analyzed together (Peñagarikano et al. 2015; Mohapatra et al. 2025; Al Abed et al. 2024). Regarding social novelty preference of Cntnap2 KO mice, only male data can be found describing no preference toward unknown conspecifics in *Cntnap2* KO mice (Levy et al. 2019). Based on our findings regarding lower density of CR+ cells and a previous study determining that absence of CR causes disruption in social novelty preference in CR KO male mice (Zhang et al. 2025), we examined how males and females perform in the social novelty test. Previously, reduced interaction time with conspecific during sociability test was reported in *Cntnap2* KO males, which could be restored by inhibiting the Akt–mTOR signaling pathway (Xing et al. 2019). Furthermore, *Cntnap2* KO male mice show altered preference in emotional state preference task and sex preference task compared to WT mice (Mohapatra et al. 2025). Here, we described an altered preference of *Cntnap2* KO mice toward the known conspecifics compared to that of the WT mice. This phenomenon can only be seen in females, suggesting a sex‐specific difference in disrupted social behavior. In accordance with previous literature data about males (Levy et al. 2019), we did not find preference toward the unknown conspecifics in male Cntnap2 KO mice.

We also examined if there were any differences in the behavior of *Cntnap2* KO and WT mice freely interacting with a same‐sex conspecific. It has been shown that *Cntnap2* KO male mice spent less time socially interacting with unknown conspecifics in the homecage reciprocal social interaction test (Selimbeyoglu et al. 2017), which can be restored upon either reducing the cortical balance (Selimbeyoglu et al. 2017) or chemogenetically or optogenetically stimulating oxytocin+ terminals in the nucleus accumbens (Choe et al. 2022). In contrast, no difference in time spent interacting with a conspecific was found in an examination using an older 12–14 weeks old cohort (Mohapatra et al. 2025). In addition, a more thorough and recent study focusing on both male and female adult *Cntnap2* KO mice showed no difference in the examined behavioral elements—body sniffing, anogenital sniffing, moving, sit idle—in the free social interaction task between KO and WT mice (Mohapatra et al. 2025). Contrary to these studies, we examined male and female adult animals in a neutral open field arena and not in their homecage. In addition, we distinguished seven different behavioral elements and found differences in the frequency, latency, and duration in “moving away,” as *Cntnap2* KO mice moved away from their partner more often, earlier, and longer.

### 4.5 Correlations Between CR+ Density and Social Withdrawal

Based on postmortem human results (Adorjan et al. 2017), we hypothesized that lower striatal CR+ interneuron density may be associated with stronger ASD‐like behavioral alterations in *Cntnap2* KO mice.

Although overall CR+ interneuron density was lower in *Cntnap2* KO mice compared to WT mice, within the KO group, greater frequency and duration of the moving away behavior were in fact correlated with higher CR+ density—a correlation absent in WT mice. These findings suggest that CR+ interneurons are associated with the persistence and the intensity of this behavior. To our knowledge, no studies have directly linked CR+ interneurons to specific behavioral components such as “moving away.” However, CR+ interneurons are known to primarily target other inhibitory interneurons, thereby exerting a disinhibitory effect and modulating excitation–inhibition balance within cortical circuits (Gulyás et al. 1996; Tremblay et al. 2016; Saffari et al. 2019). Through this mechanism, they may influence information processing and behavioral responses indirectly. Striatal CR+ neurons form molecularly and structurally distinct subpopulations (Garas et al. 2018), although their relationship with spiny projection neurons (SPNs), the dominant neuron type of the striatum (Gerfen 1992), remains unknown. SPNs can be subdivided based on dopamine receptor expression into direct‐pathway SPNs (dSPNs, D1‐expressing, movement‐initiating) and indirect‐pathway SPNs (iSPNs, D2‐expressing, movement‐suppressing) (Alexander et al. 1986; Gerfen et al. 1990). If CR+ subpopulations preferentially modulating iSPNs are selectively affected in *Cntnap2* KO mice, striatal output could shift toward more persistent dSPN‐driven signaling, amplifying social withdrawal‐related behavior. Social approach–avoidance decisions involve cortical processing of socially relevant stimuli and their integration within corticostriatal circuits that guide action selection (Yizhar et al. 2011; Balleine et al. 2007; Gerfen and Surmeier 2011). In this context, alterations in CR+ interneuron populations could contribute to changes in local inhibitory dynamics, thereby influencing social withdrawal‐related behavior. While this interpretation is consistent with canonical basal ganglia models and supported by the modulatory effects interneurons exert on SPNs (Tepper et al. 2018; Wegman et al. 2024), the specific synaptic connections of striatal CR+ interneurons and direct evidence for such subtype‐specific interactions in the context of Cntnap2 deficiency is currently lacking. Therefore, any contribution of CR+ interneurons to altered behavioral output should be interpreted with caution and within the context of broader network dysfunction.

An alternative but not mutually exclusive explanation comes from recent findings that loss of Caspr2 disrupts Kv1.2 channel clustering, selectively increasing the intrinsic excitability of dSPNs (Cording et al. 2025). In this scenario, *Cntnap2* deficiency may directly bias striatal output toward enhanced direct‐pathway activity, independent of CR+ cell density. Furthermore, disorganized clusters of Kv1.2 channels were also described in the corpus callosum and the internal capsule in *Cntnap2* KO mice (Scott et al. 2019). Disrupted Kv1.2 clustering in the corticostriatal tract suggests that striatal alterations in *Cntnap2* KO mice may partly arise from abnormal cortical input. Notably, we also observed reduced CR+ interneuron density in the nose region of the SSC, which sends topographically organized projections to the CP (Hintiryan et al. 2016).

Altogether, the findings of the present study raise the possibility that altered striatal dynamics in *Cntnap2* KO mice may emerge from the convergence of local interneuron imbalance, intrinsic dSPN hyperexcitability, and impaired cortical input processing. While the present findings regarding the association between CR+ interneurons and social withdrawal are correlational, they identify a relationship between CR+ interneuron density and behavioral output and provide a foundation for hypothesis‐driven future studies to directly test the role of CR+ interneurons in circuit‐level mechanisms underlying social withdrawal behavior.

## Funding

Institutional Excellence in Higher Education Grant (FIKP, Semmelweis University), MTA Bolyai Fellowship (BO/00277/23/5), Semmelweis Technology and Innovation Grant and Thematic Excellence Program for I.A. DKOP‐23 Doctoral Excellence Program and Gedeon Richter Excellence PhD Grant of Gedeon Richter Talentum Foundation (Gedeon Richter Plc., 1103 Budapest Gyömrői út 19‐21.) for V.S., the strategic research fund of the University of Veterinary Medicine Budapest (SRF‐002) for G.P., and NKFIH OTKA K146077 and NKKP Excellence 151425 and MTA NAP2022‐I‐3/2022 (NAP 3) for Á. D. The International Science and Technology Cooperation Projects of Guangdong Province (2023A0505050121) for I.A. and L.S., Guangdong Basic and Applied Basic Research Foundation (2022B1515130007) for L.S.

## Conflicts of Interest

The authors declare no conflicts of interest.

## Supporting information


**Figure S1:** Calretinin‐immunopositive (CR+) interneuron density in the somatosensory cortex (SSC). Overall CR+ cell density in the whole SSC (A), in the upper limb (B) and in the mouth (C) subregions showed no significant difference between WT (blue) and *Cntnap2* KO (red) mice. In the nose subregion (D) CR+ interneuron density was significantly lower in the *Cntnap2* KO group. When stratified by sex, we observed no significant difference within females or males between *Cntnap2* KO and WT mice in the overall CR+ cell density (E), upper limb (F), and mouth (G) subregions, while in the nose subregion (H) only females showed significant difference in CR+ interneuron density. Layerwise analysis revealed no significant difference in CR+ cell density in the whole SSC (I), upper limb (J), mouth (K), and nose (L) subregions.
**Table S1:** Summary table of the statistical results from linear mixed model and post hoc comparisons of calretinin‐immunopositive (CR+) interneuron density in the somatosensory cortex (SSC) between *Cntnap2* KO and wild‐type mice. The only significant (*p* < 0.05, shown in bold) difference was found in the overall CR+ cell density of the nose subregion.
**Table S2:** Summary table of statistical results from linear mixed model and post hoc comparisons of the effect of sex on calretinin‐immunopositive (CR+) interneuron density in the somatosensory cortex (SSC) between *Cntnap2* KO and wild‐type (WT) mice. Although according to the LMM the effect of sex on CR+ cell density was not significant, but sex stratified analysis revealed a significant difference between *Cntnap2* KO and WT animals only within the female group.
**Figure S2:** Parvalbumin‐immunopositive (PV+) interneuron density in the caudoputamen (CP). Analysis of overall PV+ neuron density (A, *p* = 0.3519) and sex‐stratified PV+ cell density (B, *p* = 0.8886 for females and *p* = 0.1530 for males) in the caudoputamen of *Cntnap2* KO (red) and WT (blue) mice revealed no significant difference between the two experimental groups.
**Figure S3:** Parvalbumin‐immunopositive (PV+) interneuron density in the somatosensory cortex (SSC). Overall PV+ cell density in the whole SSC (A), in the upper limb (B), in the mouth (C), and in the nose (D) subregions showed no significant difference between wild‐type (WT, blue) and *Cntnap2* KO (red) mice. When stratified by sex, we observed no significant difference within females or males between *Cntnap2* KO and WT mice in the overall PV+ cell density (E), upper limb (F), mouth (G), and nose (H) subregions. Layerwise analysis revealed no significant difference in CR+ cell density in the whole SSC (I), upper limb (J), mouth (K), and nose (L) subregions.
**Table S3:** Summary table of the statistical results from linear mixed model and post hoc comparisons of parvalbumin‐immunopositive (PV+) interneuron density in the somatosensory cortex (SSC) between *Cntnap2* KO and wild‐type mice. No significant difference was found in the overall CR+ cell density or in any examined subregions.
**Table S4:** Summary table of statistical results from linear mixed model and post hoc comparisons of the effect of sex on parvalbumin‐immunopositive interneuron density in the somatosensory cortex between *Cntnap2* KO and wild‐type mice.
**Figure S4:** Area of the caudoputamen (CP) and thickness of the somatosensory cortex (SSC) in Cntnap2 KO and WT mice. No significant difference was found in the area of the CP (A, *p* = 0.1862) or the thickness of the SSC (B, *p* = 0.7255).
**Table S5:** Summary of the statistical results of the comparison of *Cntnap2* KO and wild‐type control groups during social novelty test. Unpaired two‐tailed Student's *t*‐test pairwise comparisons were applied to reveal significant differences in the time spent in each chamber. Significant values (*p* < 0.05) are shown in bold.
**Table S6:** Summary of *p*‐values from unpaired two‐tailed Student's *t*‐test pairwise comparisons of time spent in each chamber during the social novelty test. Significant values (*p* < 0.05) are shown in bold.
**Table S7:** Summary of the statistical results of the comparison of activity and total traveled distance of *Cntnap2* KO and wild‐type control groups. Unpaired two‐tailed Student's *t*‐test pairwise comparisons were applied to reveal significant differences in the time spent in each chamber. Significant values (*p* < 0.05) are shown in bold.
**Table S8:** *p*‐Values for the effect of group, sex, and their interaction on behavior elements. Linear regression model was applied. Significant values (*p* < 0.05) are shown in bold.
**Table S9:** *p*‐Values for the effect of calretinin‐immunopositive (CR+) interneuron densities in the caudoputamen on moving away behavior. Linear regression model was applied. Significant values (*p* < 0.05) are shown in bold.
**Table S10:** *p*‐Values for the effect of calretinin‐immunopositive (CR+) interneuron densities in the somatosensory cortex on moving away behavior. Linear regression model was applied. Significant values (*p* < 0.05) are shown in bold.

## Data Availability

The data that support the findings of this study are available from the corresponding author upon reasonable request.
