# Supplementary material for: Lower Striatal and Cortical Calretinin Interneuron Density Associated With Altered Social Behavior in Cntnap2 Knockout Mice
Source: Autism Res. 2026 Jun 6;19(7):e70286. doi: 10.1002/aur.70286 (PMC13377339; doi:10.1002/aur.70286)
Supplement: Supplementary file 1 — Figure S1: Calretinin‐immunopositive (CR+) interneuron density in the somatosensory cortex (SSC). Overall CR+ cell density in the whole SSC (A), in the upper limb (B) and in the mouth (C) subregions showed no significant difference between WT (blue) and Cntnap2 KO (red) mice. In the nose subregion (D) CR+ interneuron density was significantly lower in the Cntnap2 KO group. When stratified by sex, we observed no significant difference within females or males between Cntnap2 KO and WT mice in the overall CR+ cell density (E), upper limb (F), and mouth (G) subregions, while in the nose subregion (H) only females showed significant difference in CR+ interneuron density. Layerwise analysis revealed no significant difference in CR+ cell density in the whole SSC (I), upper limb (J), mouth (K), and nose (L) subregions. Table S1: Summary table of the statistical results from linear mixed model and post hoc comparisons of calretinin‐immunopositive (CR+) interneuron density in the somatosensory cortex (SSC) between Cntnap2 KO and wild‐type mice. The only significant (p < 0.05, shown in bold) difference was found in the overall CR+ cell density of the nose subregion. Table S2: Summary table of statistical results from linear mixed model and post hoc comparisons of the effect of sex on calretinin‐immunopositive (CR+) interneuron density in the somatosensory cortex (SSC) between Cntnap2 KO and wild‐type (WT) mice. Although according to the LMM the effect of sex on CR+ cell density was not significant, but sex stratified analysis revealed a significant difference between Cntnap2 KO and WT animals only within the female group. Figure S2: Parvalbumin‐immunopositive (PV+) interneuron density in the caudoputamen (CP). Analysis of overall PV+ neuron density (A, p = 0.3519) and sex‐stratified PV+ cell density (B, p = 0.8886 for females and p = 0.1530 for males) in the caudoputamen of Cntnap2 KO (red) and WT (blue) mice revealed no significant difference between the two experimental grou [file AUR-19-0-s001.docx]

**Calretinin**

**Figure S1.** Calretinin-immunopositive (CR+) interneuron density in the somatosensory cortex (SSC). Overall CR+ cell density in the whole SSC (A), in the upper limb (B) and in the mouth (C) subregions showed no significant difference between WT (blue) and *Cntnap2* KO (red) mice. In the nose subregion (D) CR+ interneuron density was significantly lower in the *Cntnap2* KO group. When stratified by sex, we observed no significant difference within females or males between *Cntnap2* KO and WT mice in the overall CR+ cell density (E), upper limb (F) and mouth (G) subregions, while in the nose subregion (H) only females showed significant difference in CR+ interneuron density. Layerwise analysis revealed no significant difference in CR+ cell density in the whole SSC (I), upper limb (J), mouth (K) and nose (L) subregions.


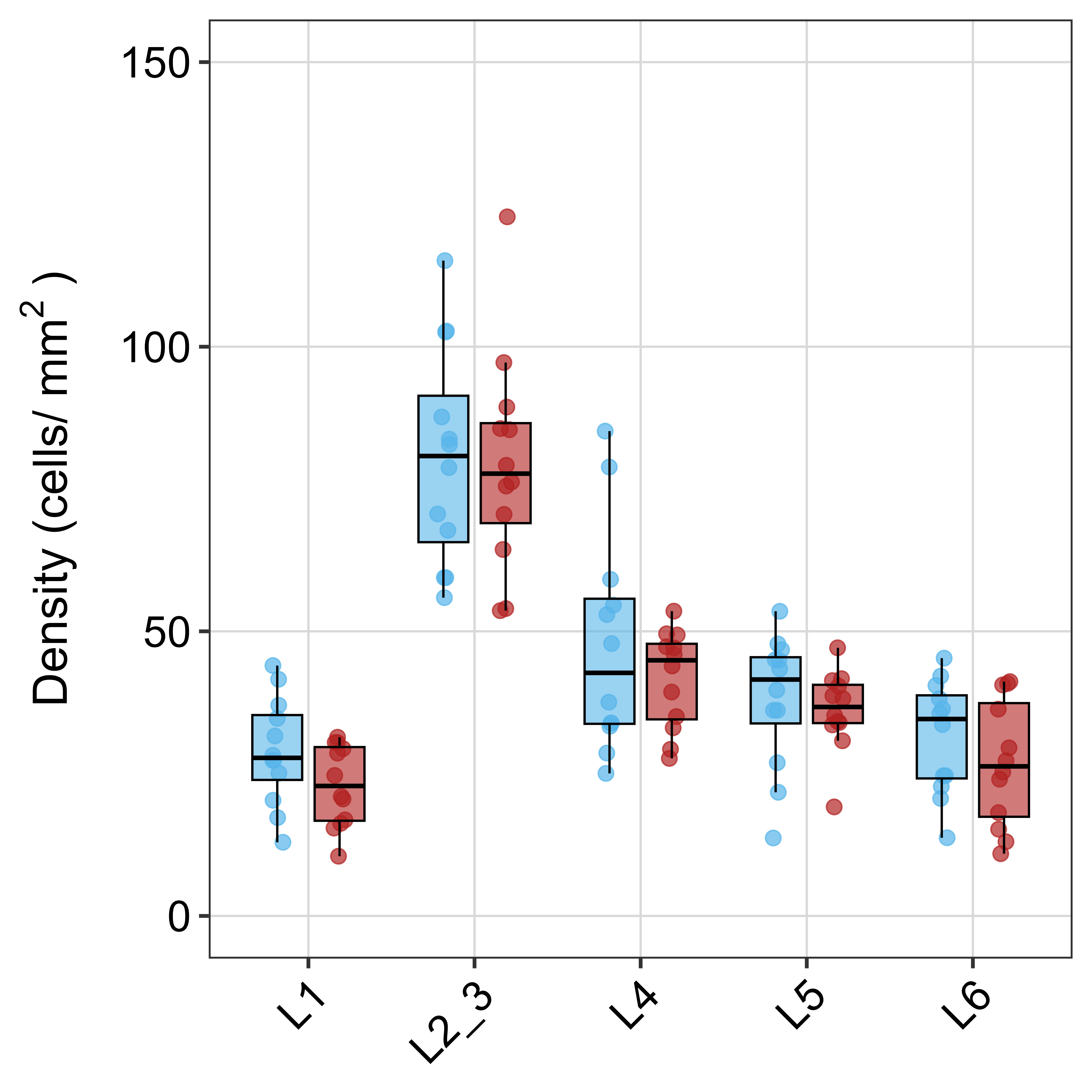

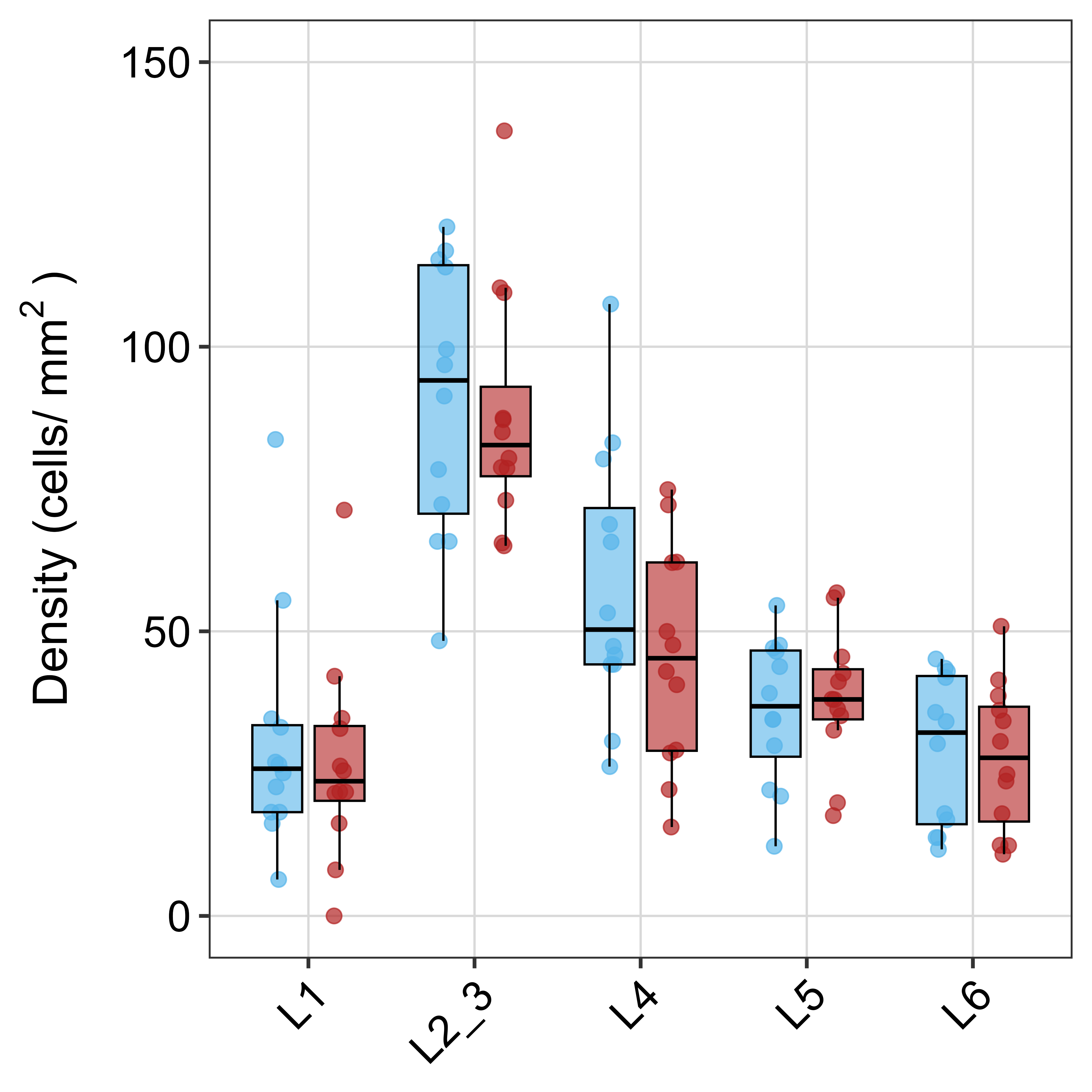

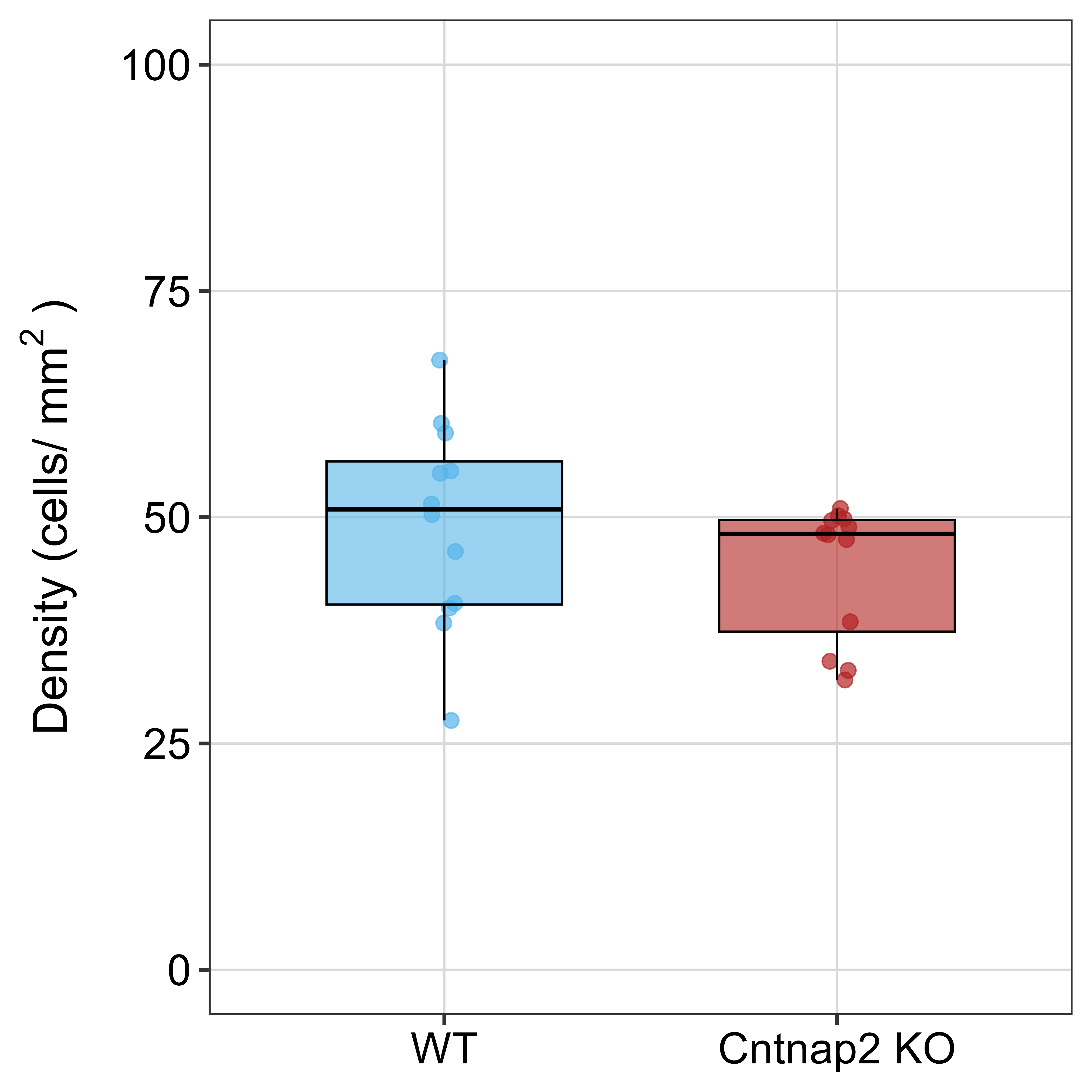

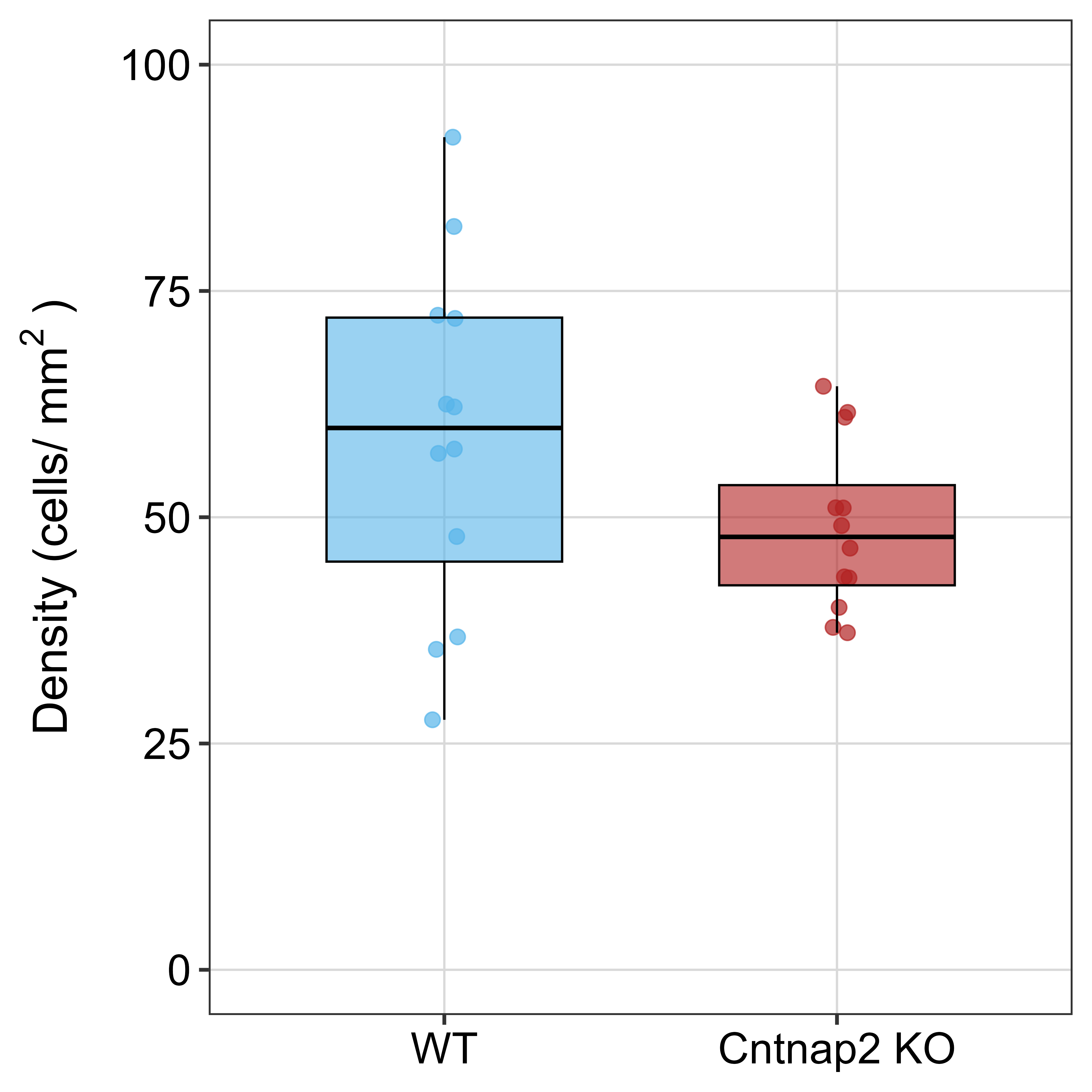

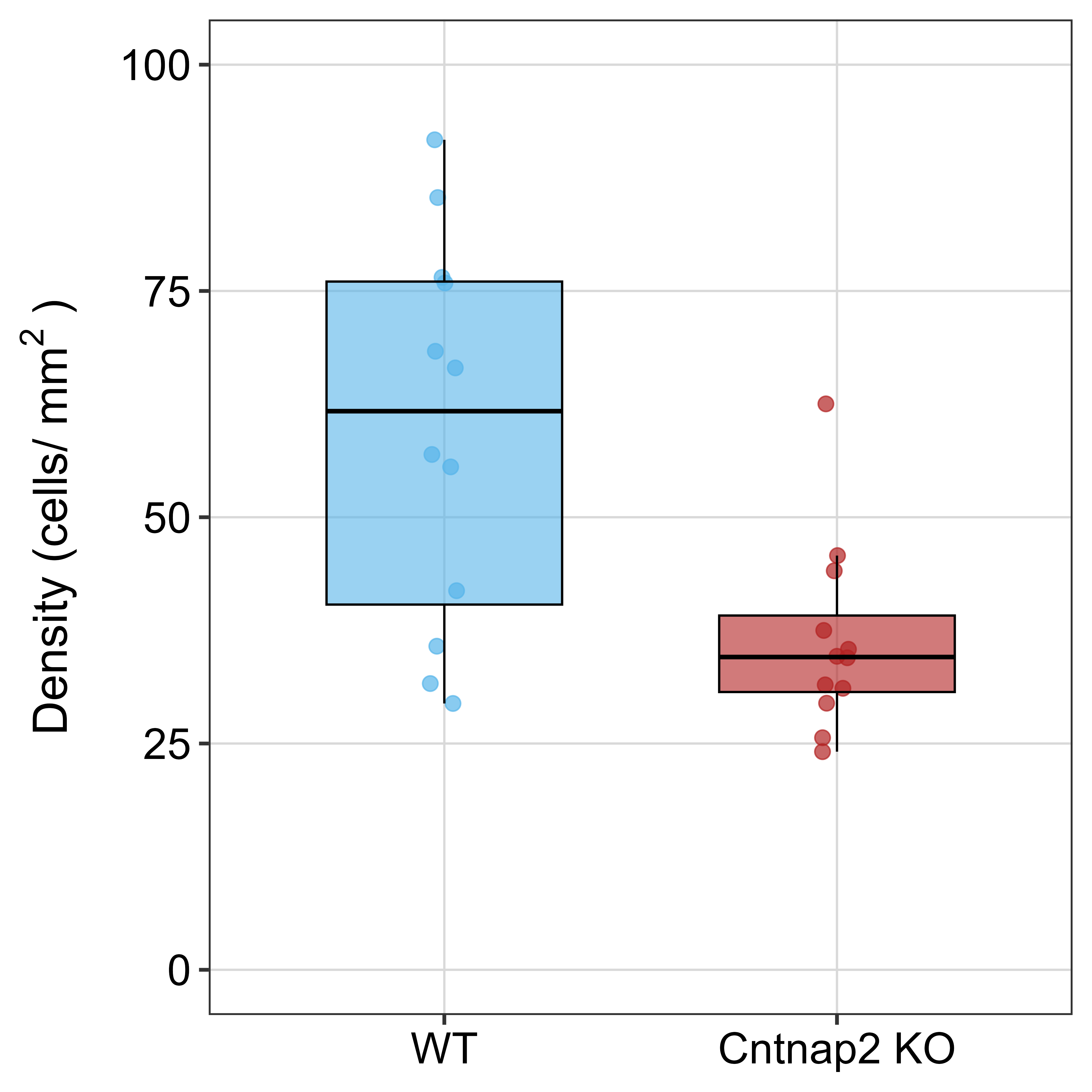

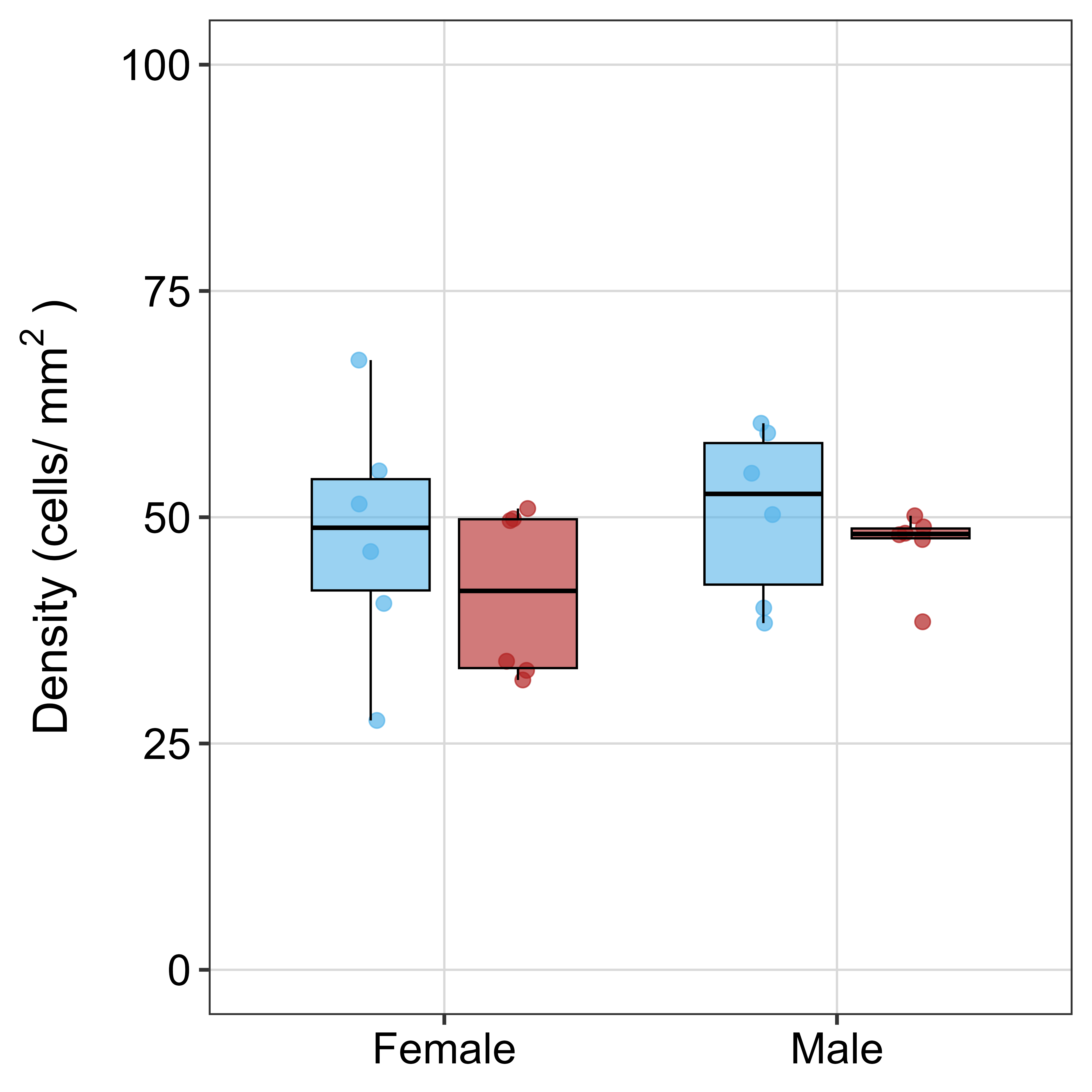

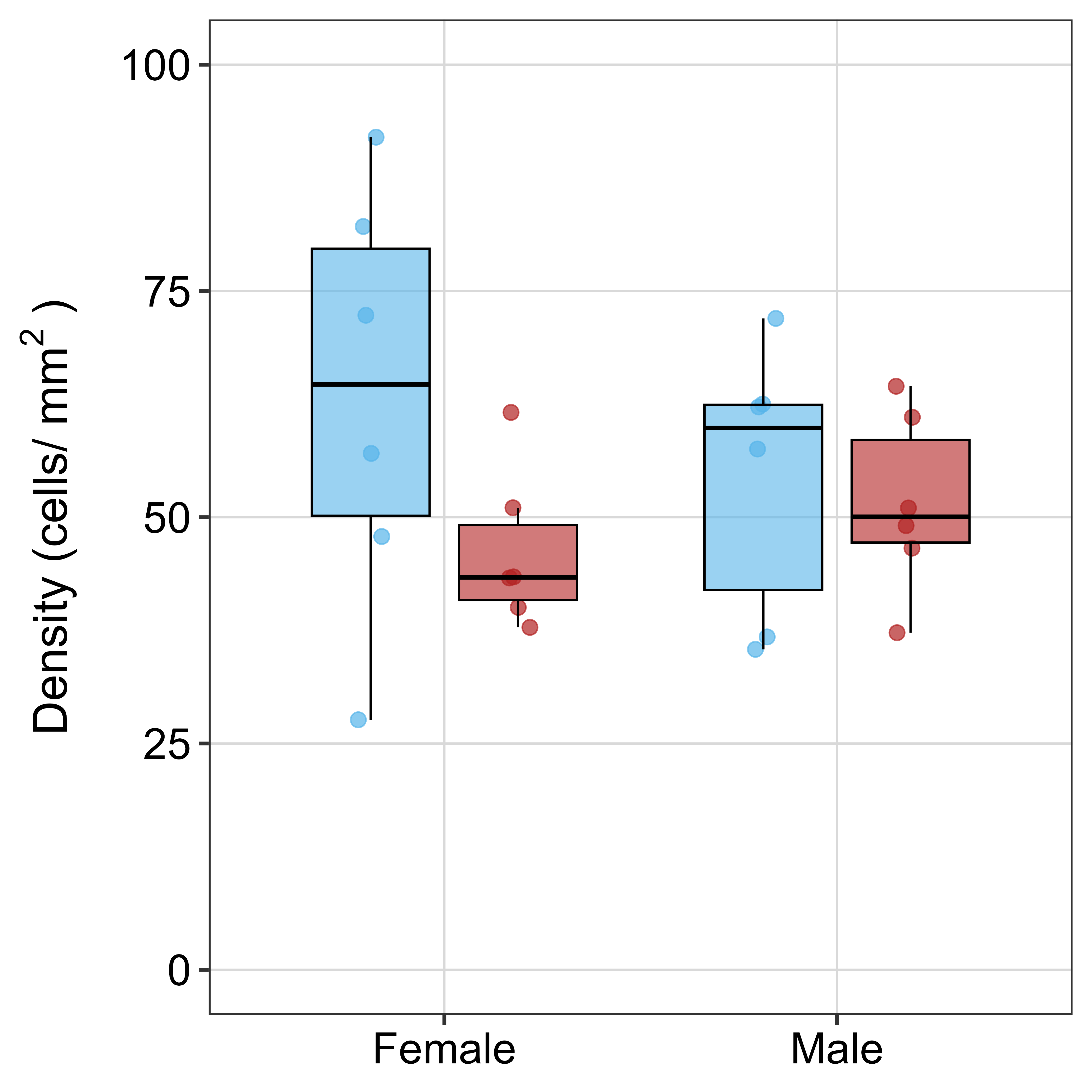

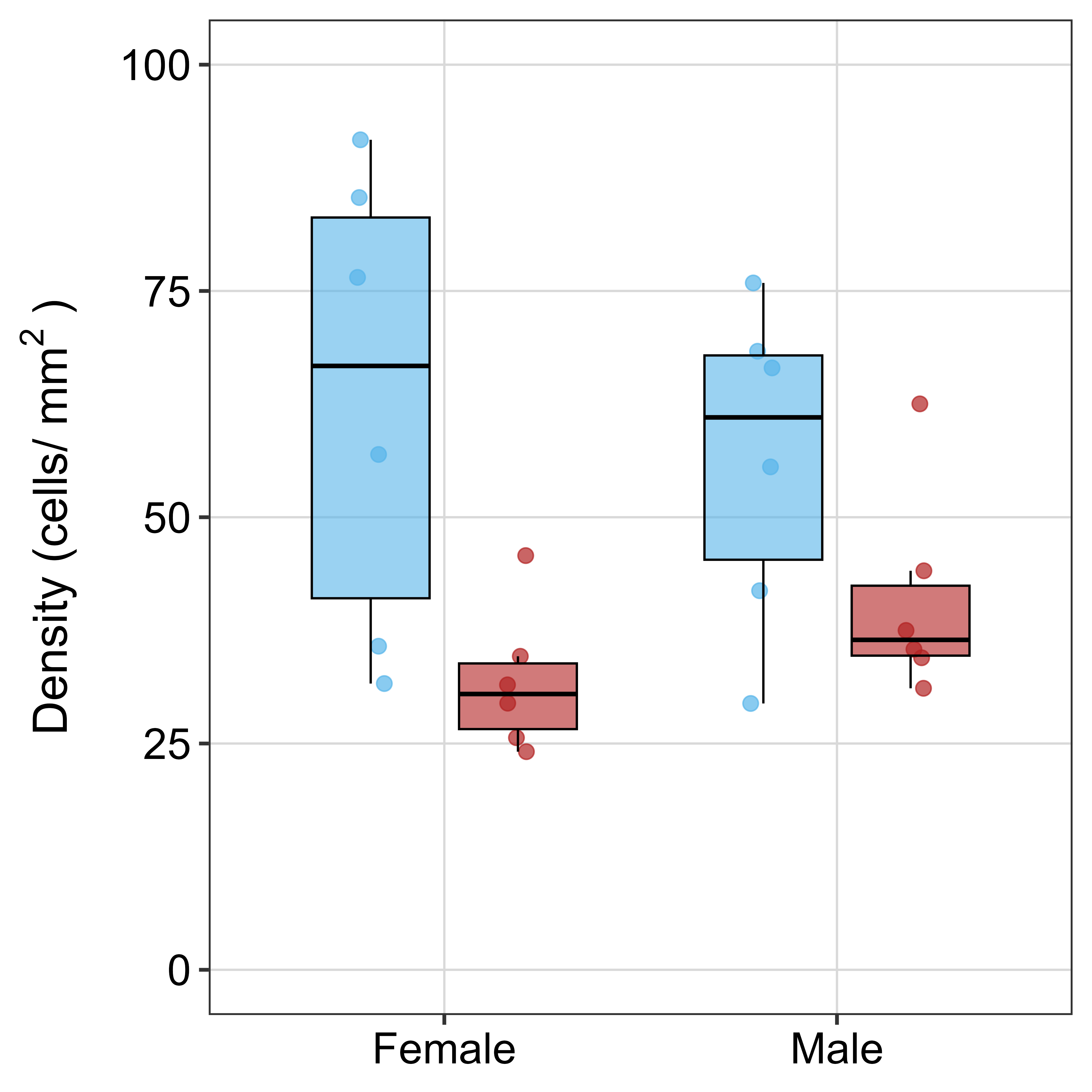

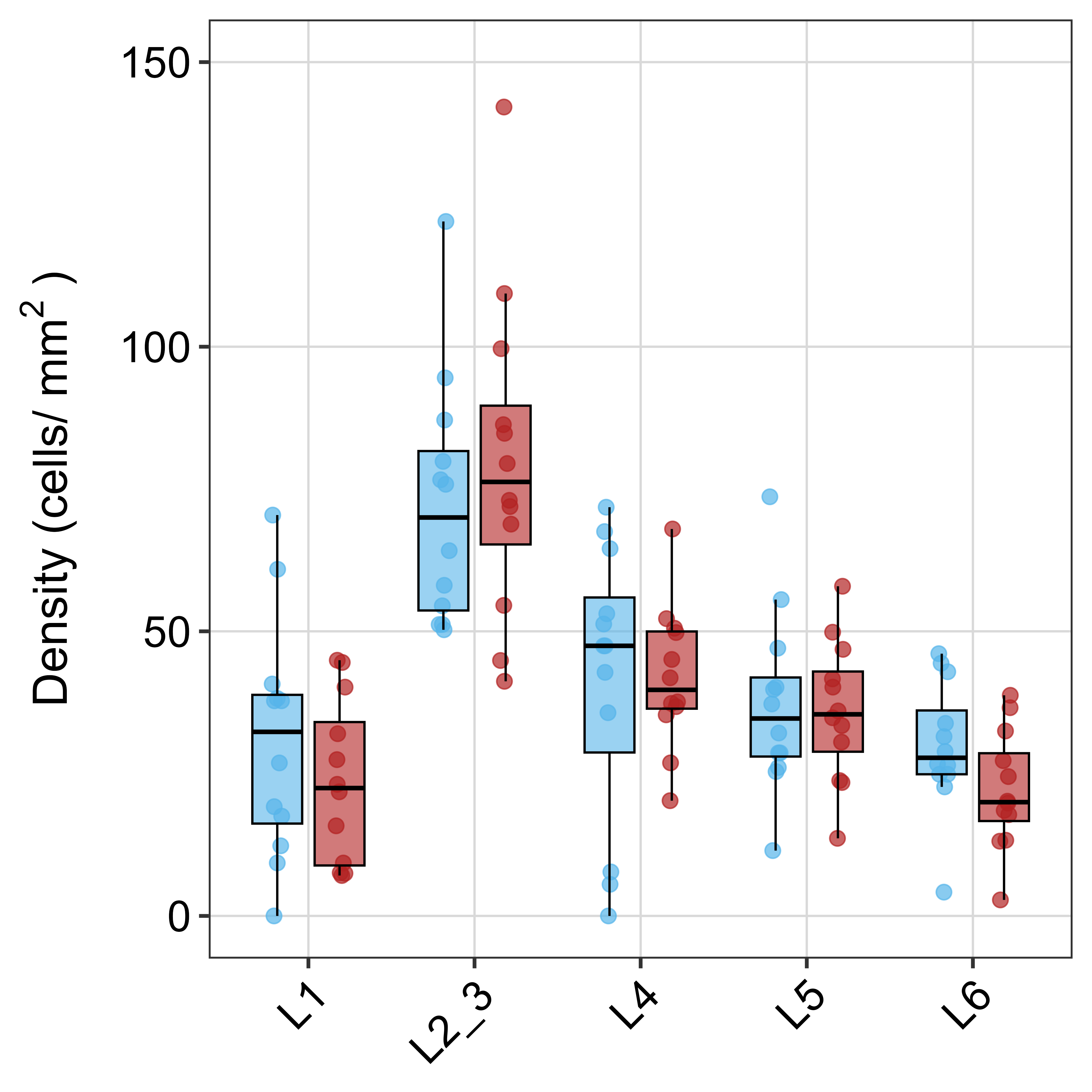

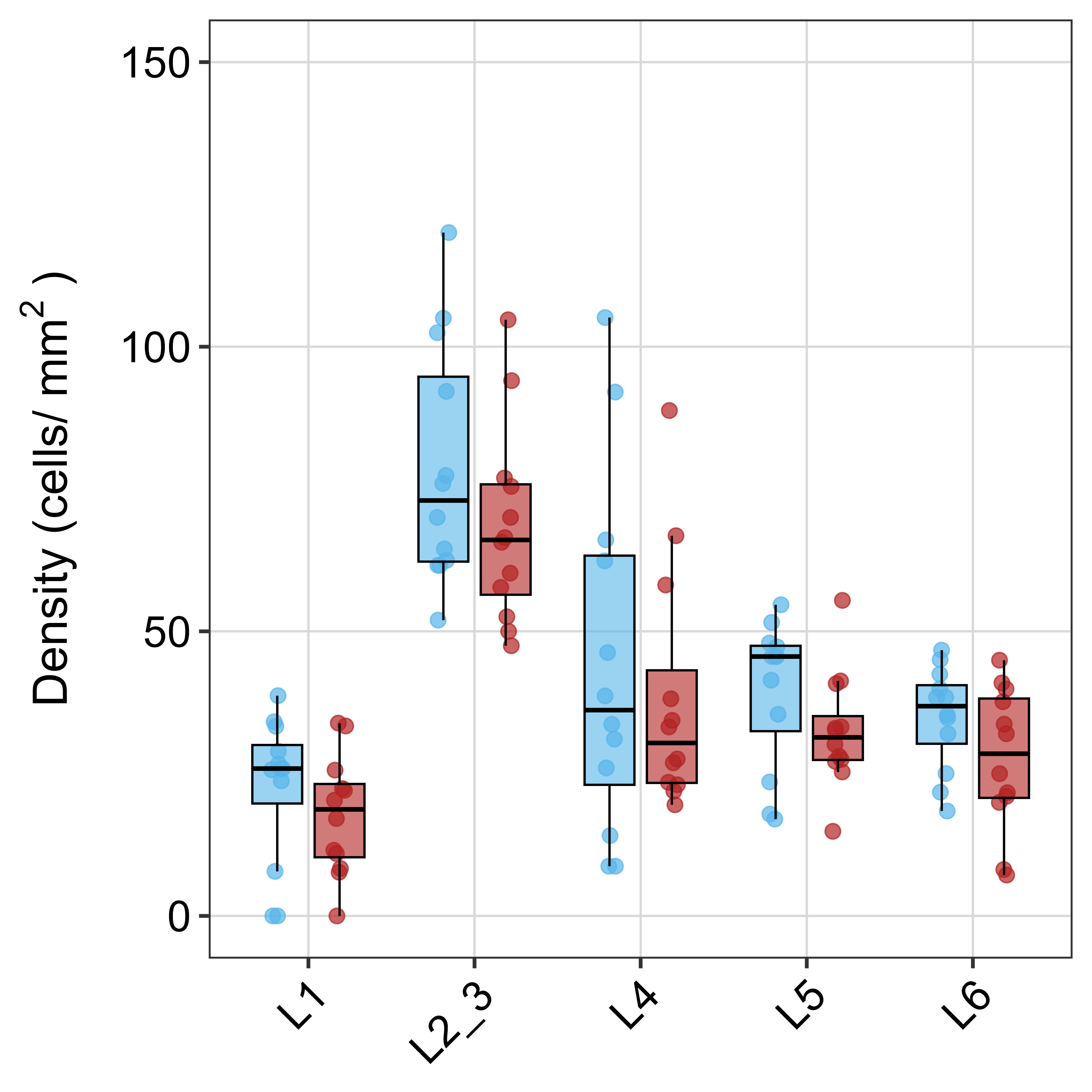


A

B

D

E

F

H

I

J

K

L

**

*


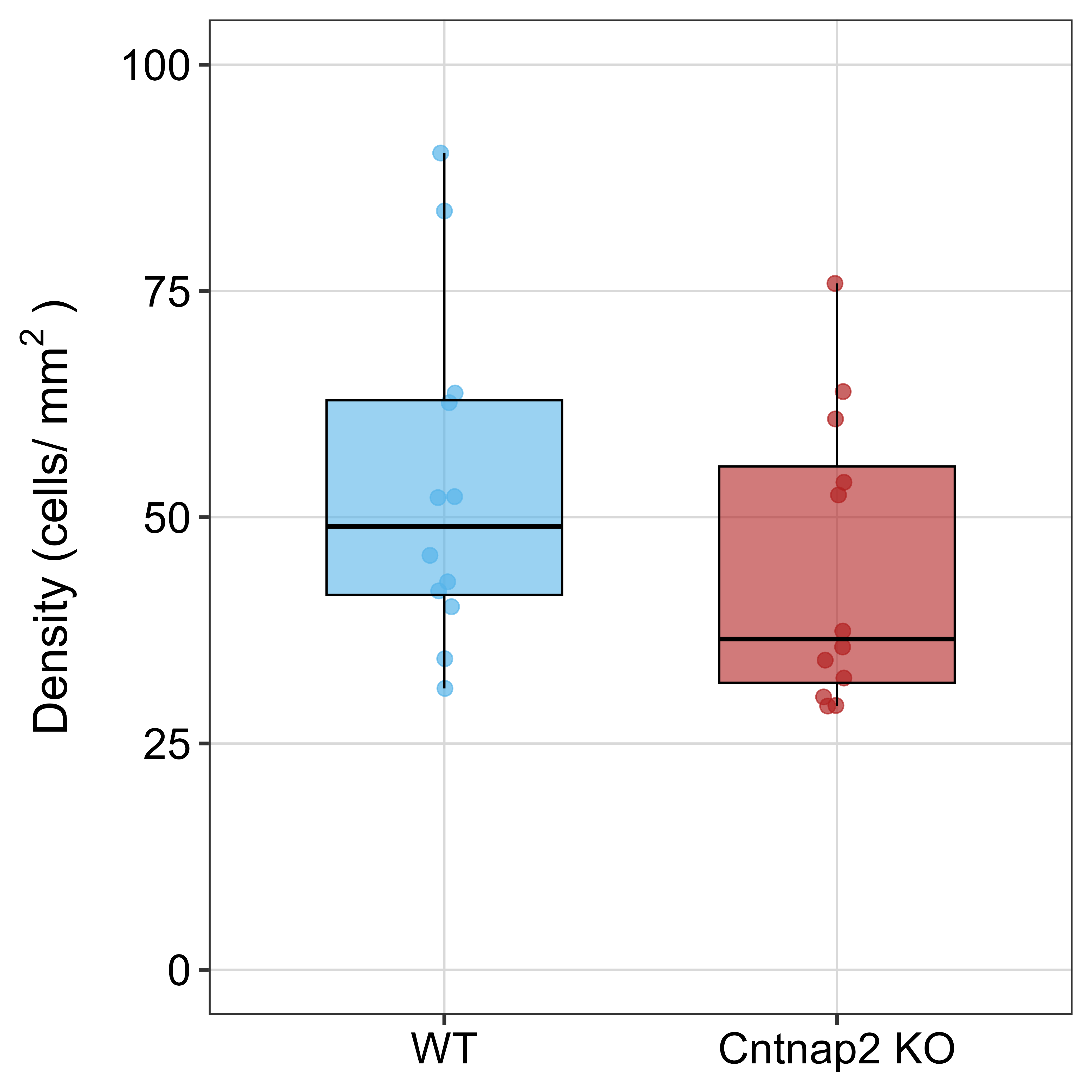


C


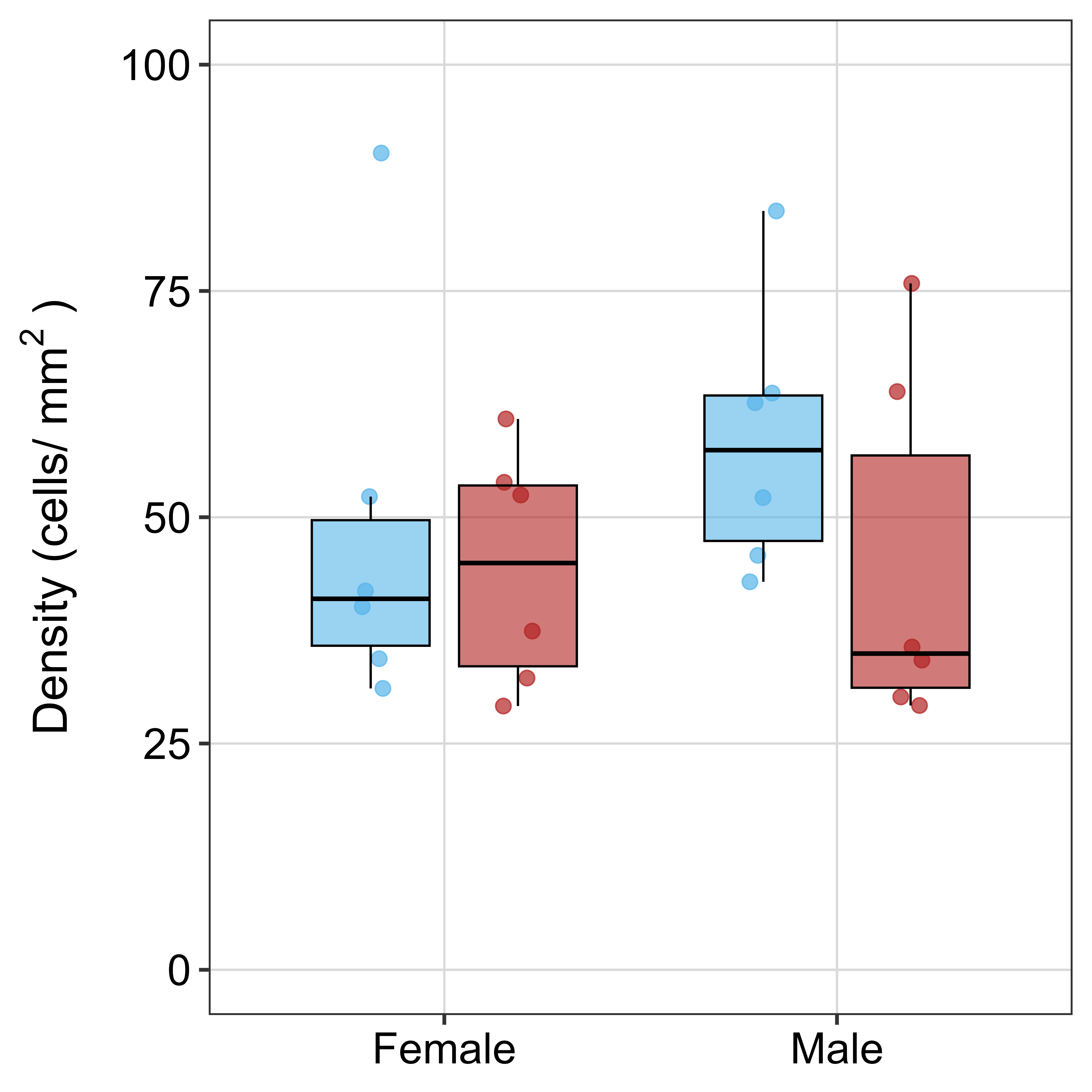


G

| **Whole somatosensory cortex** | | | | |
| --- | --- | --- | --- | --- |
| **Layer** | **p-value** | **df** | **t** | **95% CI** |
| **L1** | 0.2770 | 61.7 | 1.10 | [−4.9, 16.9] |
| **L2/3** | 0.8475 | 61.7 | 0.19 | [−9.9, 12.0] |
| **L4** | 0.2902 | 61.7 | 1.07 | [−5.1, 16.7] |
| **L5** | 0.7446 | 61.7 | 0.33 | [−9.1, 12.7] |
| **L6** | 0.3986 | 61.7 | 0.85 | [−6.3, 15.6] |
| **All layers** | 0.2109 | 21 | −1.29 | [−13.1, 3.1] |
| **Mouth subregion of SSC** | | | | |
| **Layer** | **p-value** | **df** | **t** | **95% CI** |
| **L1** | 0.3079 | 72.1 | 1.03 | [−7.0, 22.0] |
| **L2/3** | 0.3028 | 72.1 | −1.04 | [−22.1, 7.0] |
| **L4** | 0.9391 | 72.1 | −0.08 | [−15.1, 14.5] |
| **L5** | 0.8761 | 72.1 | 0.16 | [−13.4, 15.6] |
| **L6** | 0.2950 | 72.1 | 1.06 | [−6.8, 22.2] |
| **All layers** | 0.1977 | 21 | −1.23 | [−23.7, 6.1] |
| **Nose subregion of SSC** | | | | |
| **Layer** | **p-value** | **df** | **t** | **95% CI** |
| **L1** | 0.5131 | 94.1 | 0.66 | [−9.7, 19.3] |
| **L2/3** | 0.1607 | 94.1 | 1.41 | [−4.2, 24.9] |
| **L4** | 0.4200 | 94.1 | 0.81 | [−8.6, 20.4] |
| **L5** | 0.3387 | 94.1 | 0.96 | [−7.5, 21.5] |
| **L6** | 0.3276 | 94.1 | 0.98 | [−7.3, 21.7] |
| **All layers** | **0.0032** | 21 | −3.33 | [−37.9, −8.7] |
| **Upper limb subregion of SSC** | | | | |
| **Layer** | **p-value** | **df** | **t** | **95% CI** |
| **L1** | 0.6189 | 81.1 | 0.50 | [−11.2, 18.8] |
| **L2/3** | 0.7682 | 81.1 | 0.30 | [−12.8, 17.2] |
| **L4** | 0.1026 | 81.1 | 1.65 | [−2.6, 27.4] |
| **L5** | 0.7679 | 81.1 | −0.30 | [−17.2, 12.8] |
| **L6** | 0.8808 | 81.1 | 0.15 | [−13.9, 16.1] |
| **All layers** | 0.1350 | 21 | −1.56 | [−23.1, 3.3] |

**Table S1.** Summary table of the statistical results from linear mixed model and post hoc comparisons of calretinin-immunopositive (CR+) interneuron density in the somatosensory cortex (SSC) between *Cntnap2* KO and wild type mice. The only significant (p < 0.05, shown in bold) difference was found in the overall CR+ cell density of the nose subregion.

|  | **Males** | **Females** | **Sex×Genotye** | **df** | **t** | **95% CI** |
| --- | --- | --- | --- | --- | --- | --- |
| **Whole SSC** | 0.4146 | 0.3615 | 0.7281 | 21 | 1.05 | [−4.9, 14.9] |
| **Mouth** | 0.2089 | 0.7082 | 0.3666 | 21 | 1.01 | [−9.4, 27.2] |
| **Nose** | 0.1033 | **0.0171** | 0.2717 | 21 | 2.71 | [5.5, 41.1] |
| **Upperlimb** | 0.7080 | 0.1312 | 0.2770 | 21 | 1.27 | [−6.3, 26.1] |

**Table S2.** Summary table of statistical results from linear mixed model and post hoc comparisons of the effect of sex on calretinin-immunopositive (CR+) interneuron density in the somatosensory cortex (SSC) between *Cntnap2* KO and wild type (WT) mice. Although according to the LMM the effect of sex on CR+ cell density was not significant, but sex stratified analysis revealed a significant difference between *Cntnap2* KO and WT animals only within the female group.

**Parvalbumin**


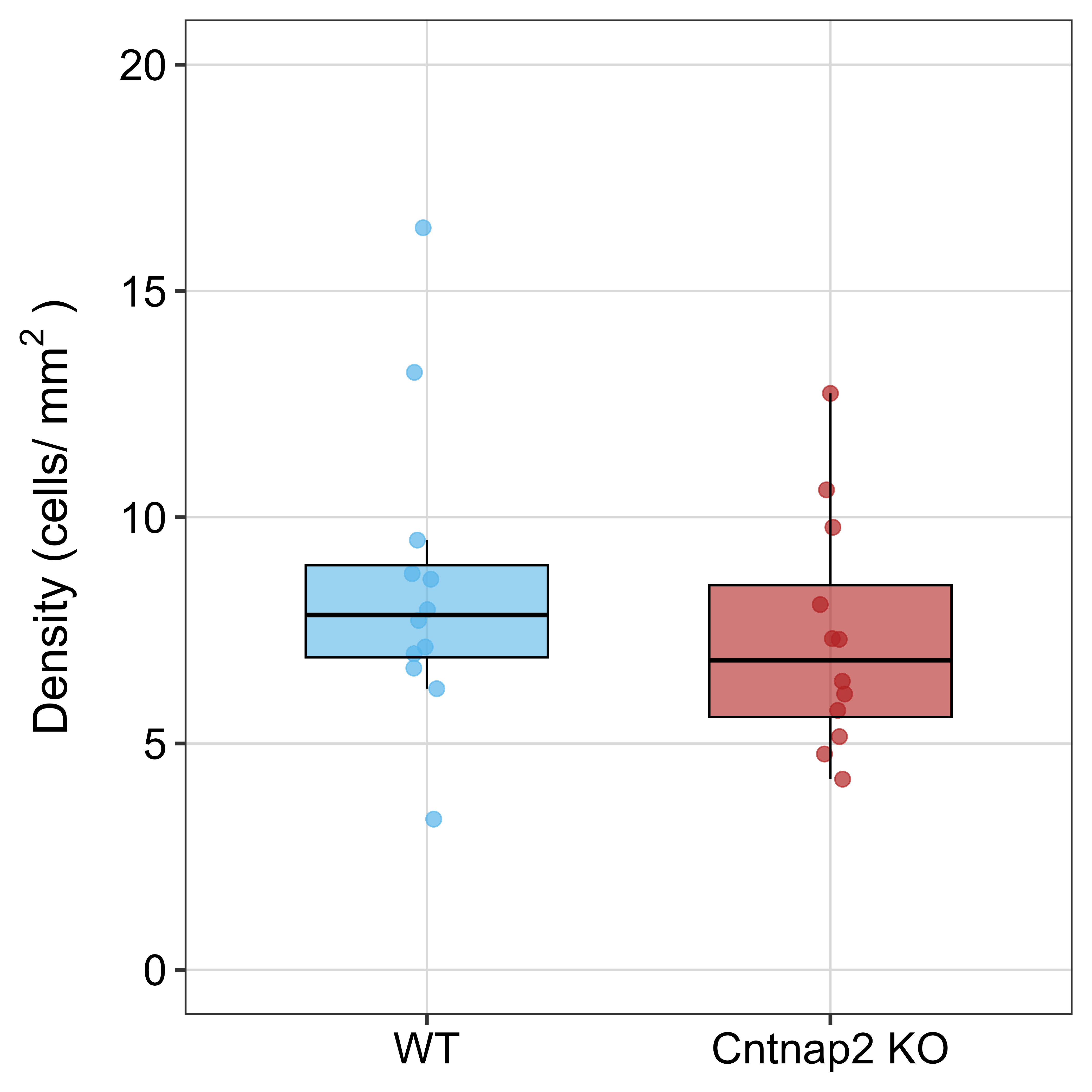

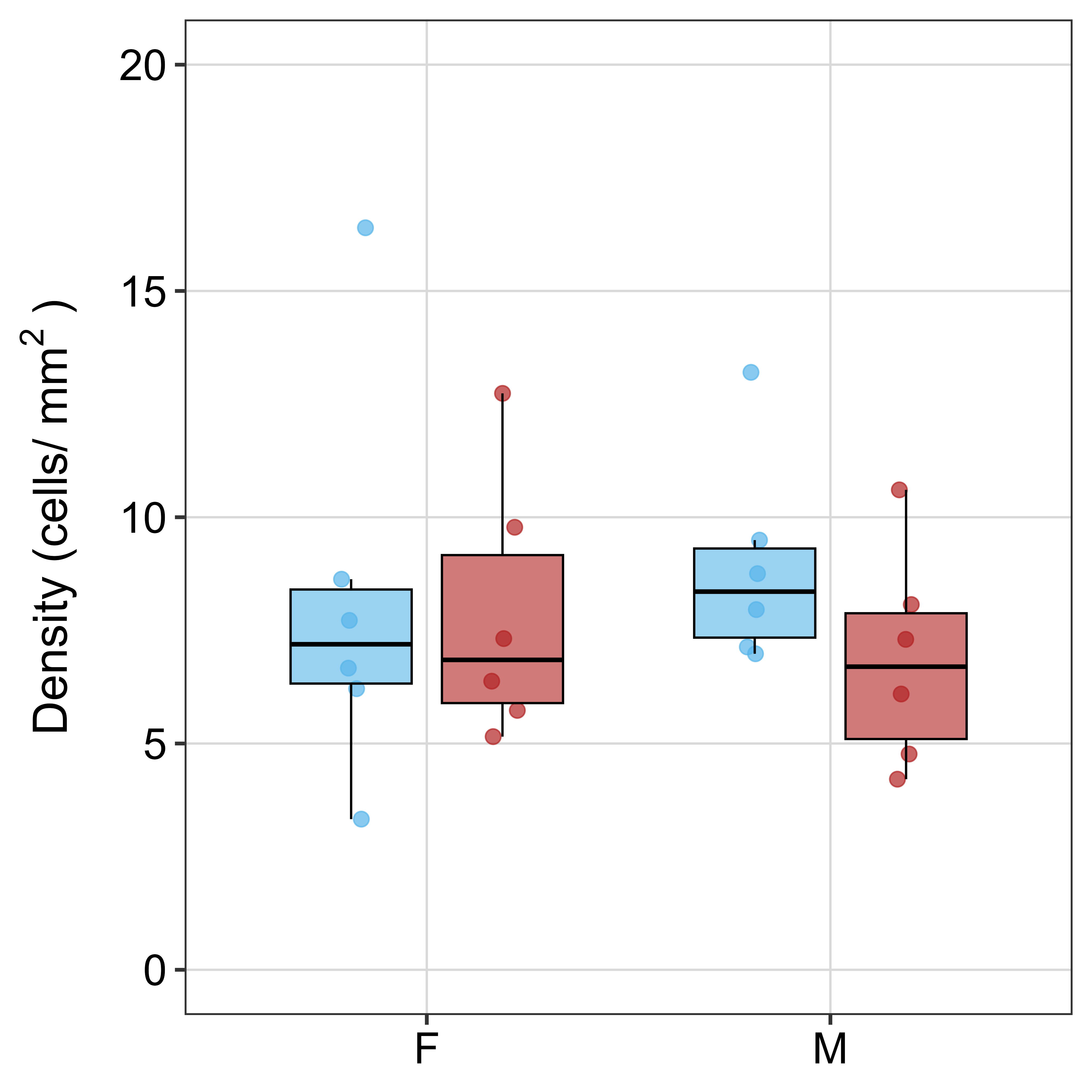


A

B

**Figure S2. Parvalbumin-immunopositive (PV+) interneuron density in the caudoputamen (CP).** Analysis of overall PV+ neuron density (A, p = 0.3519) and sex-stratified PV+ cell density (B, p = 0.8886 for females and p = 0.1530 for males) in the caudoputamen of *Cntnap2* KO (red) and WT (blue) mice revealed no significant difference between the two experimental groups.


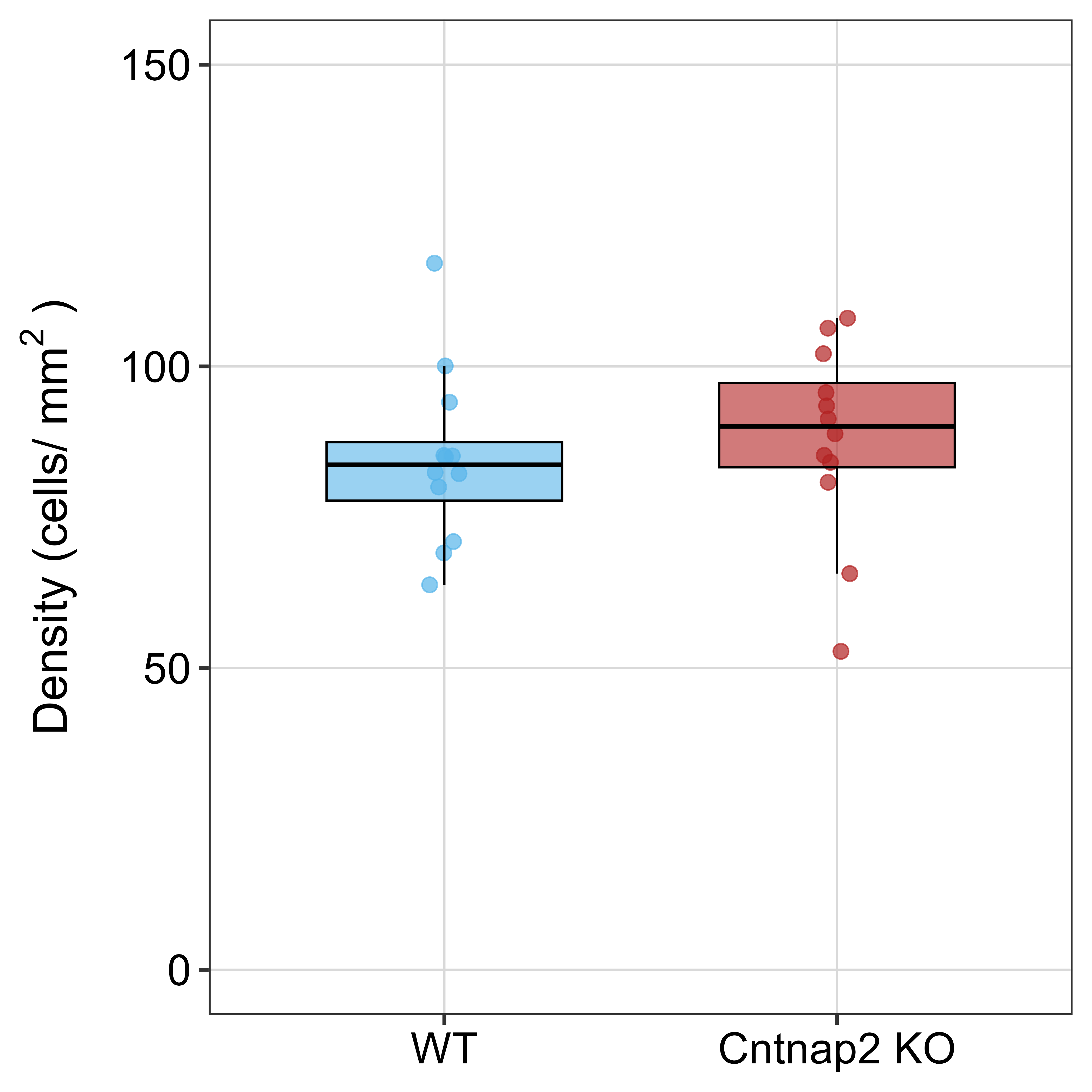

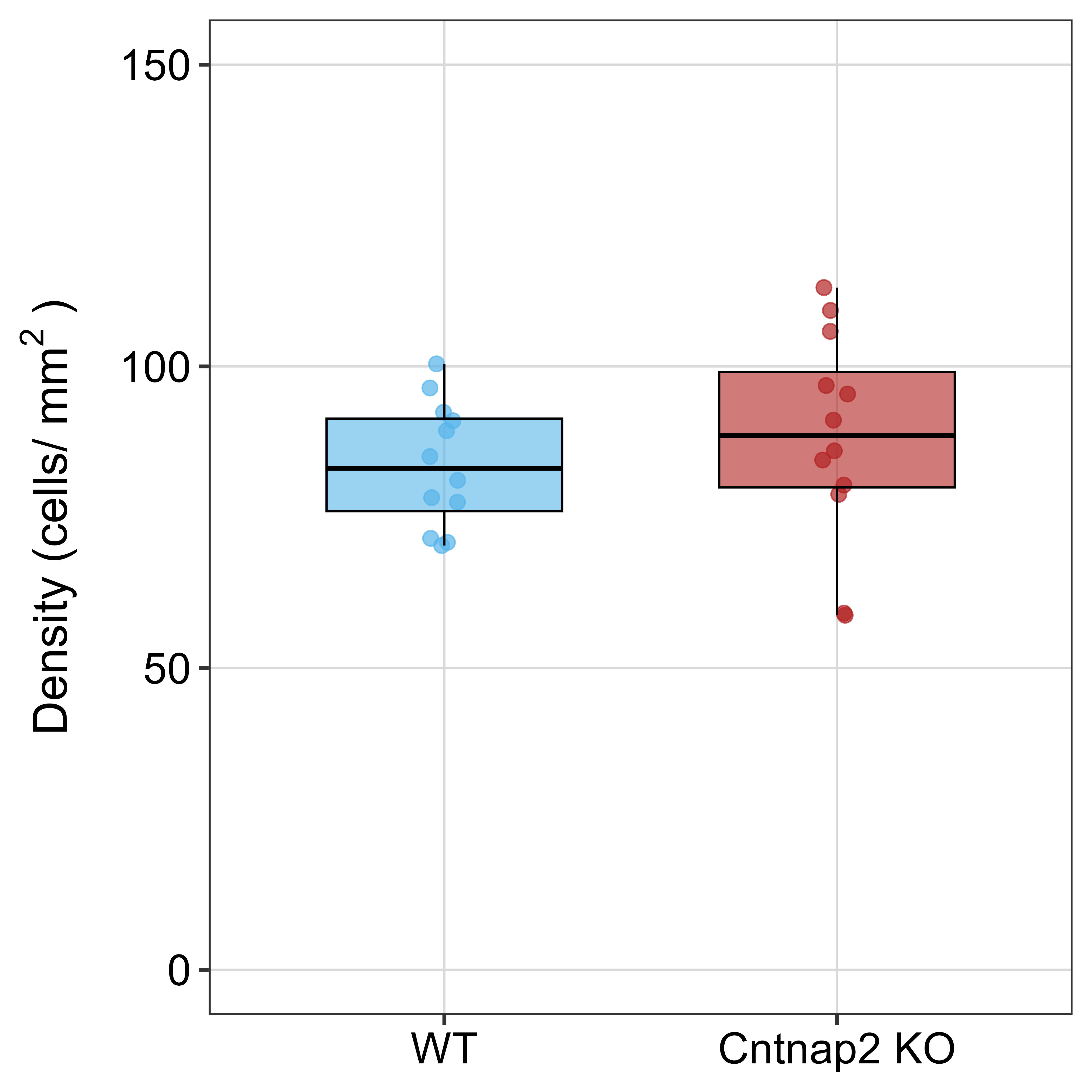

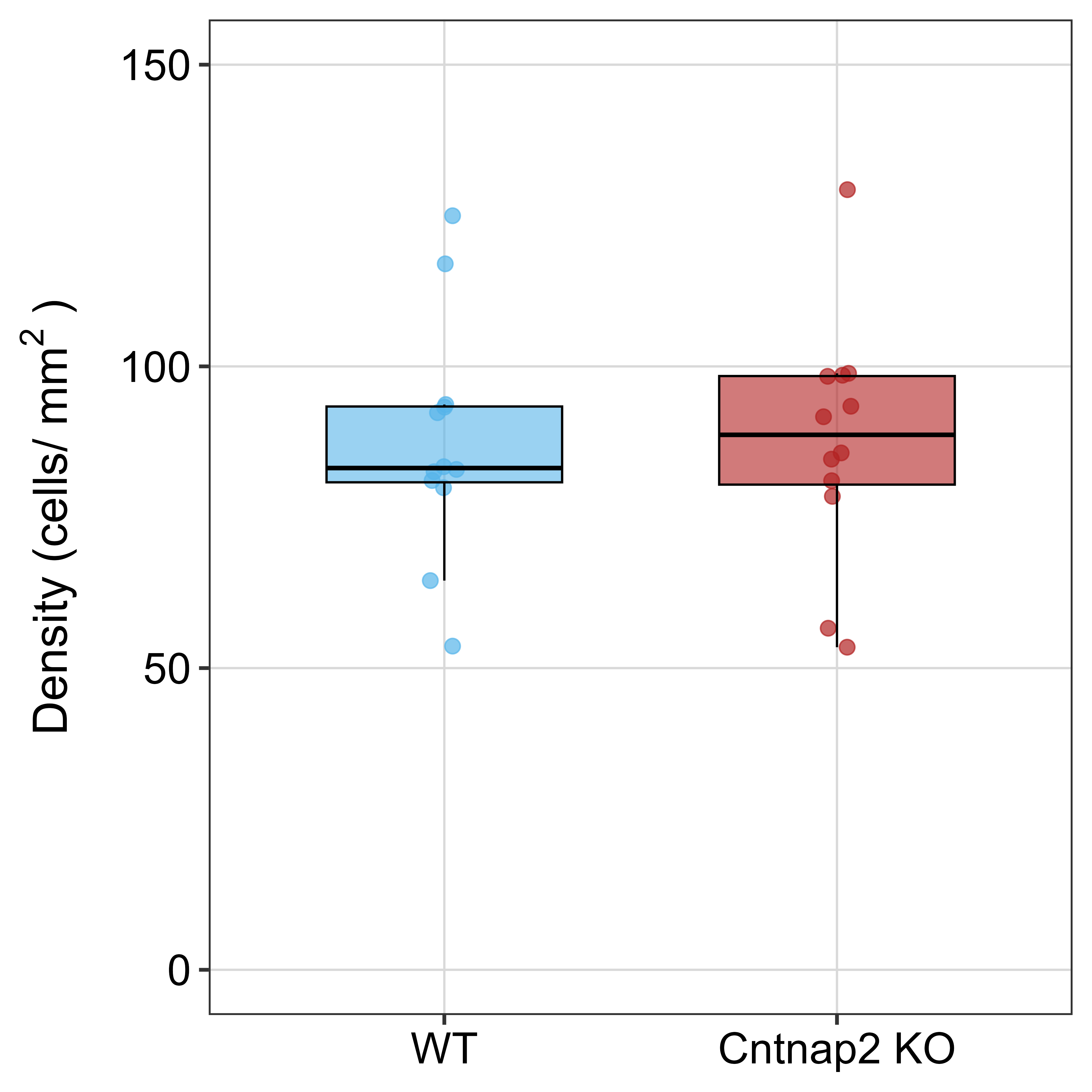

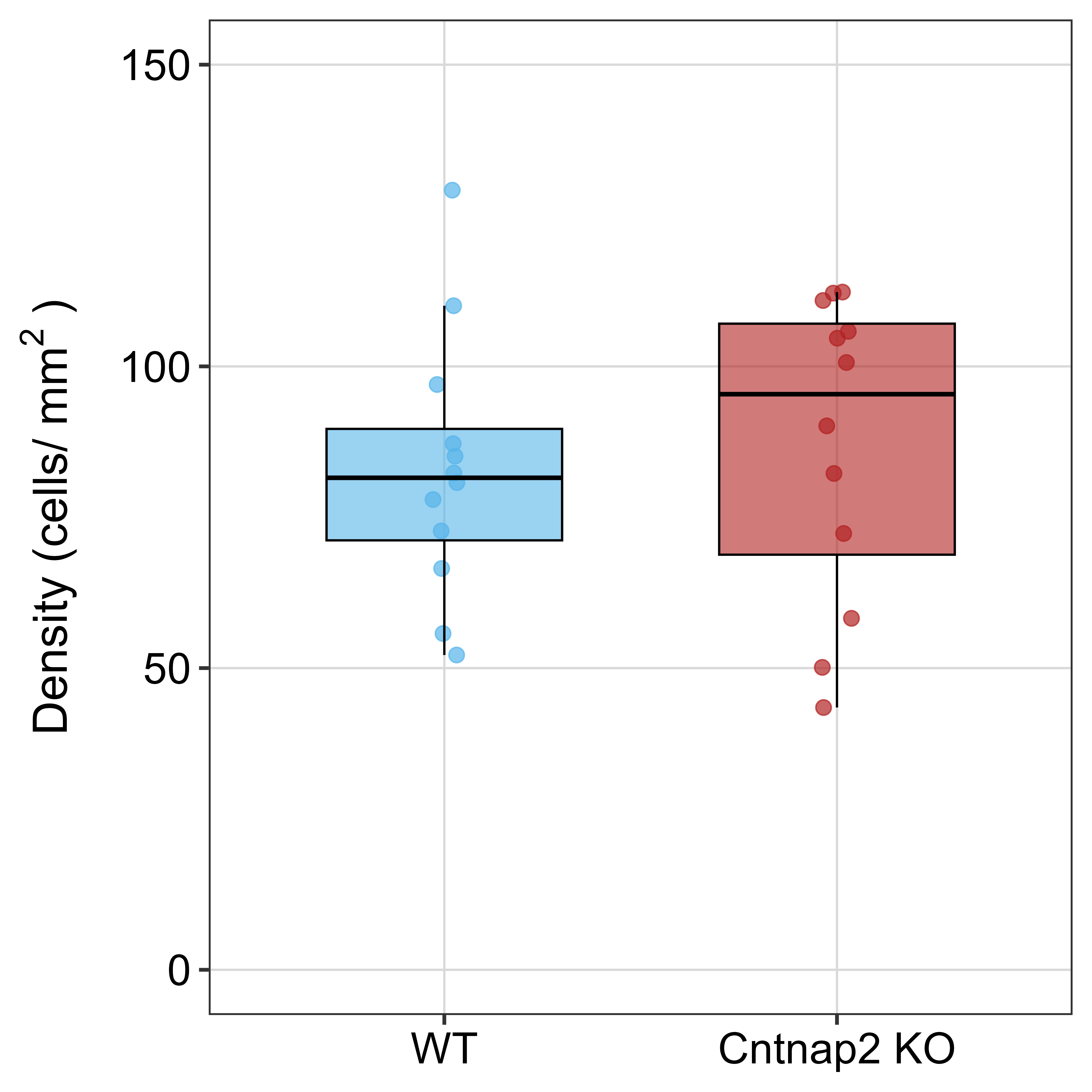

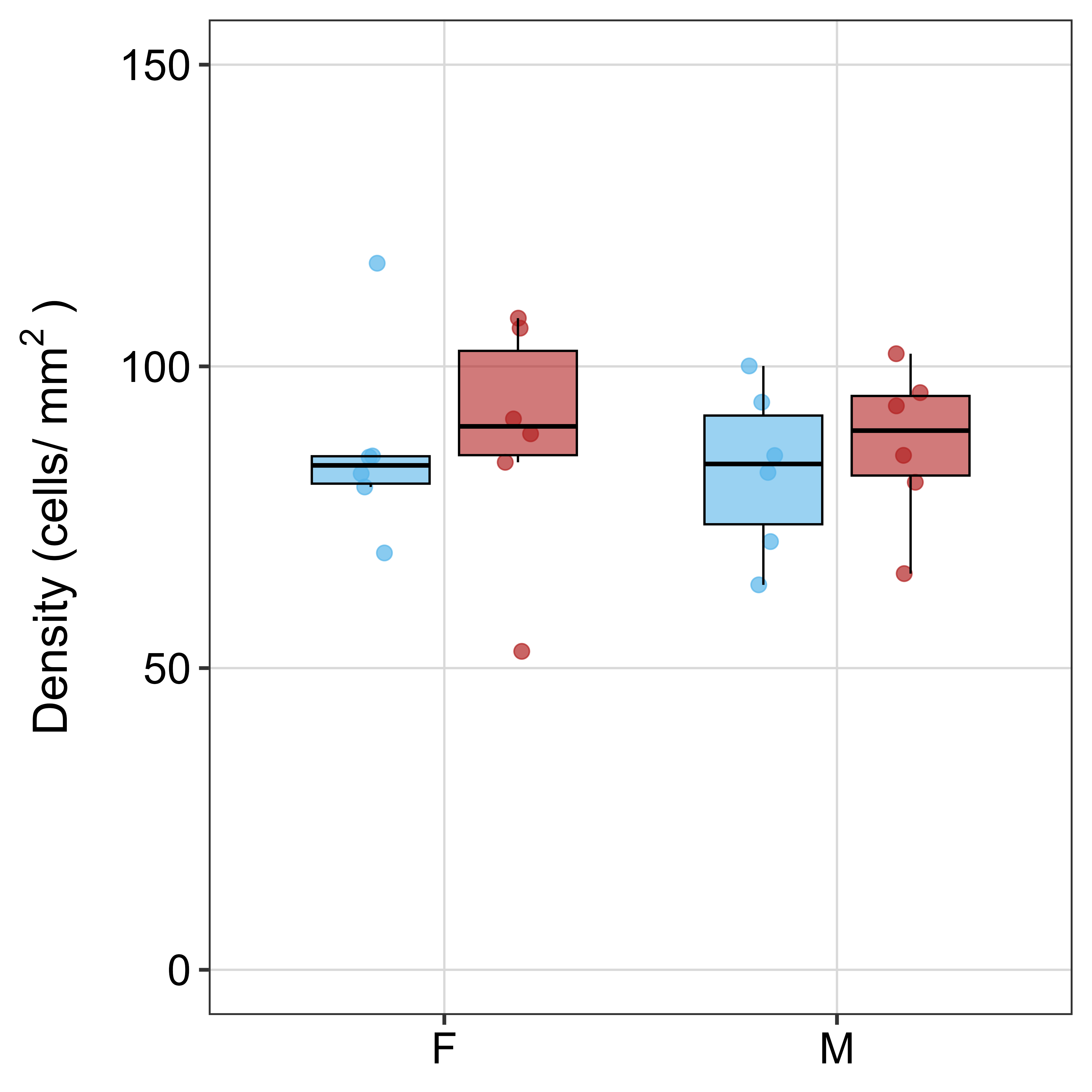

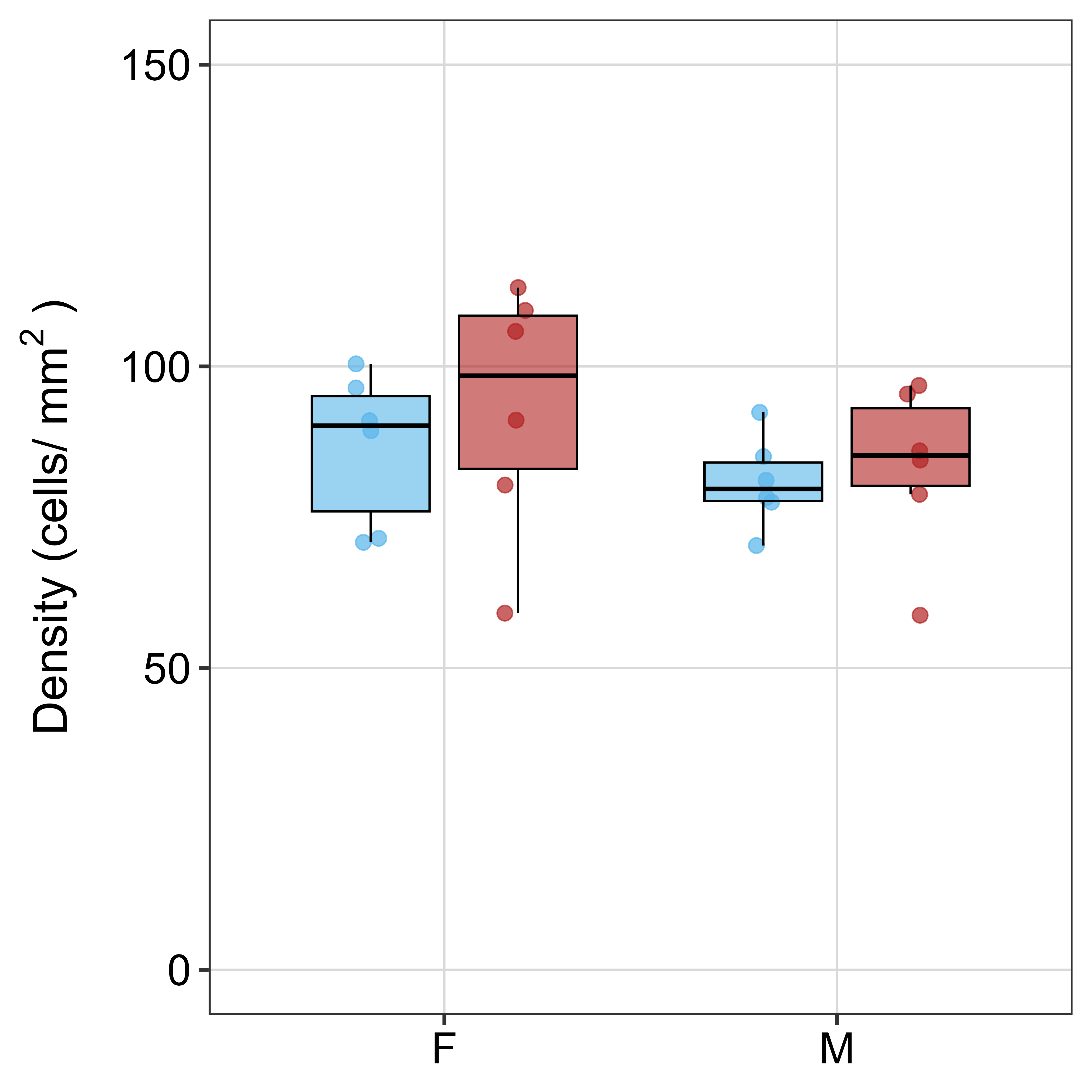

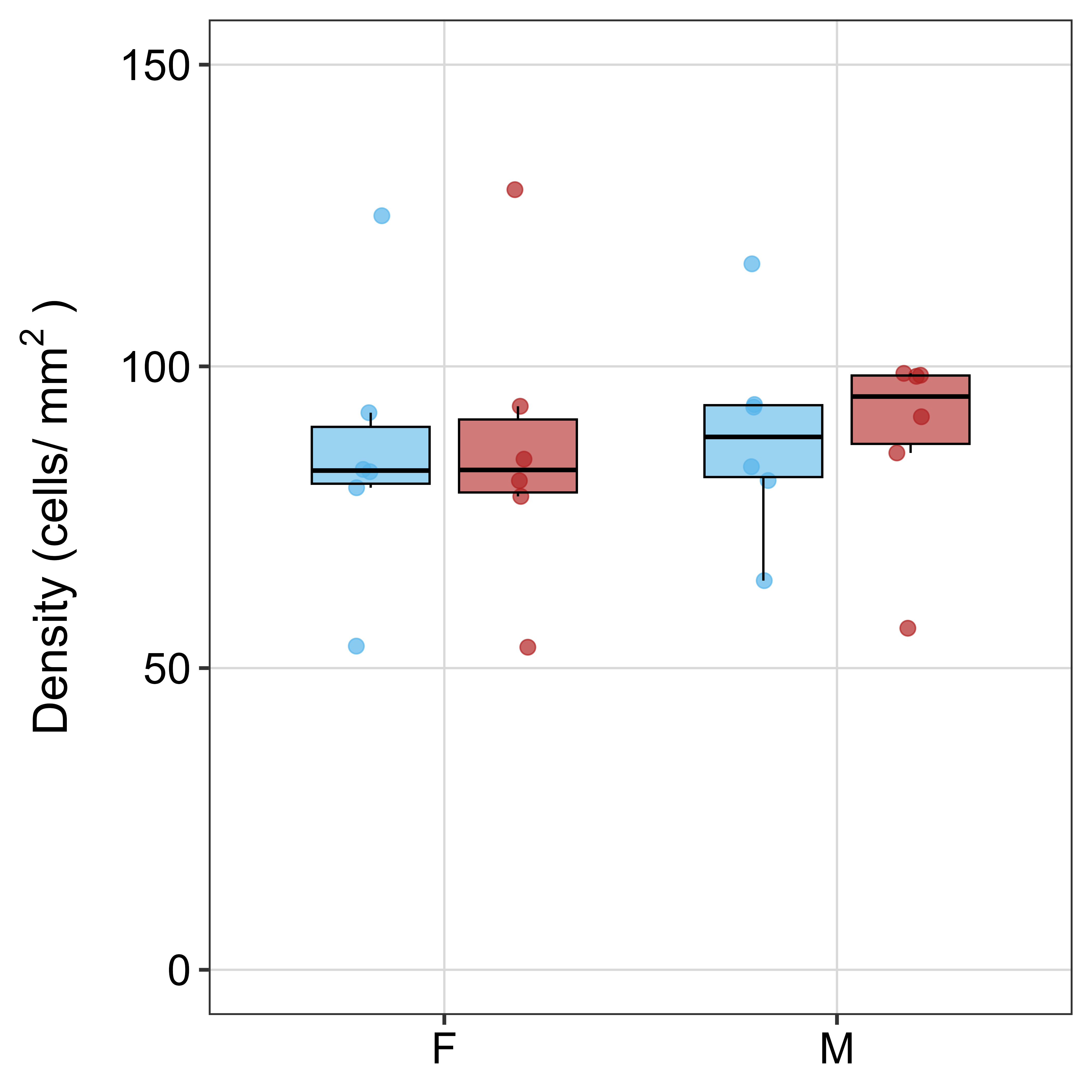

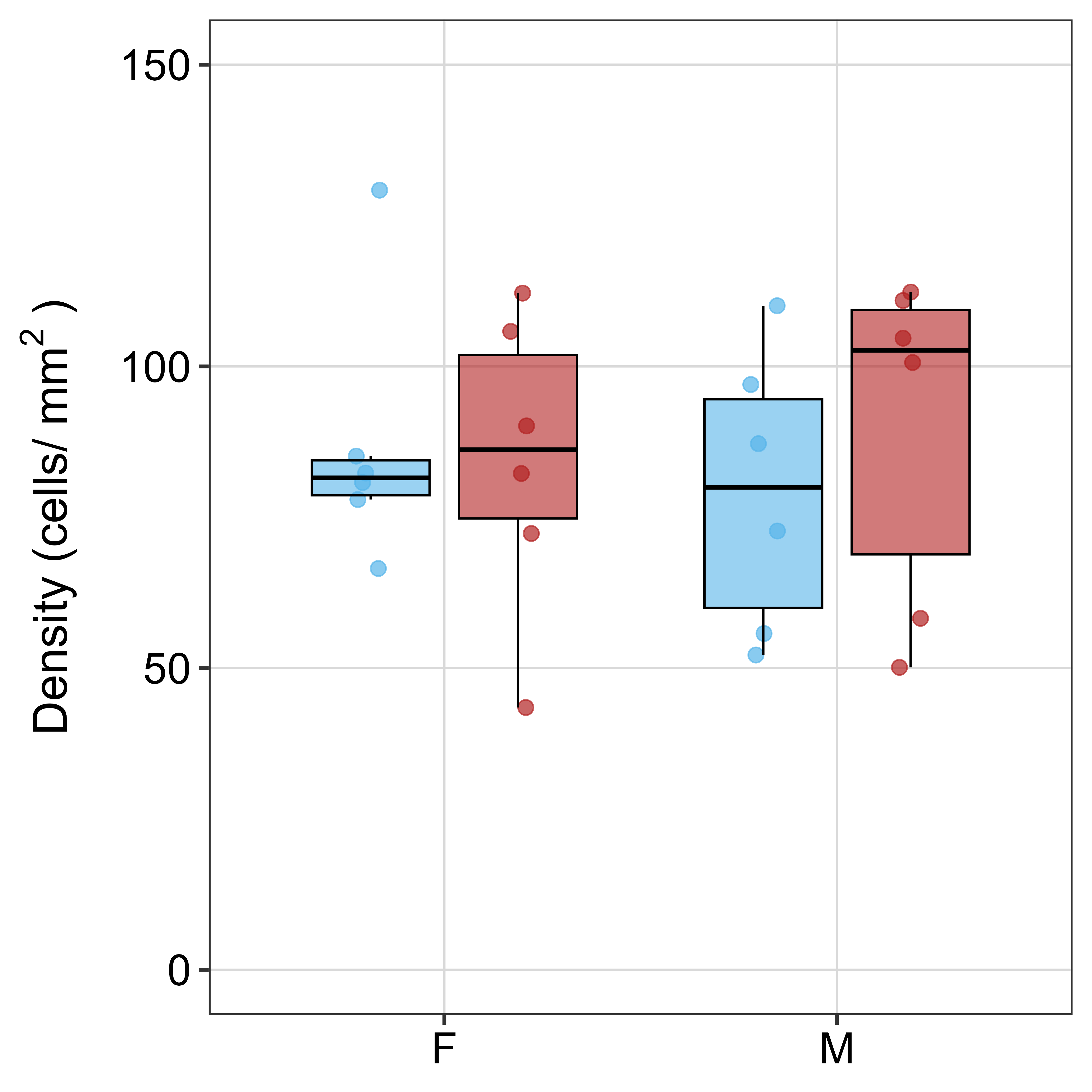

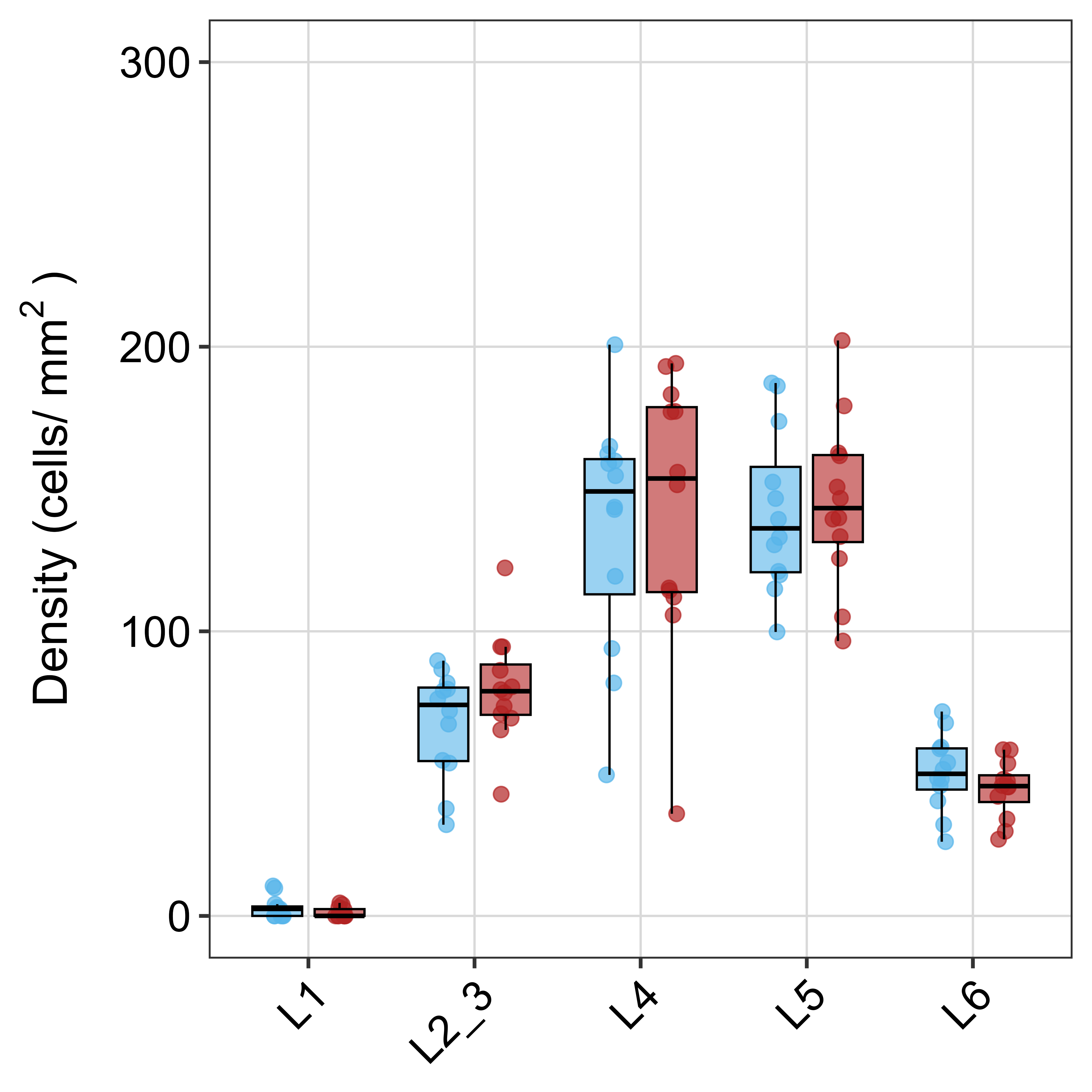

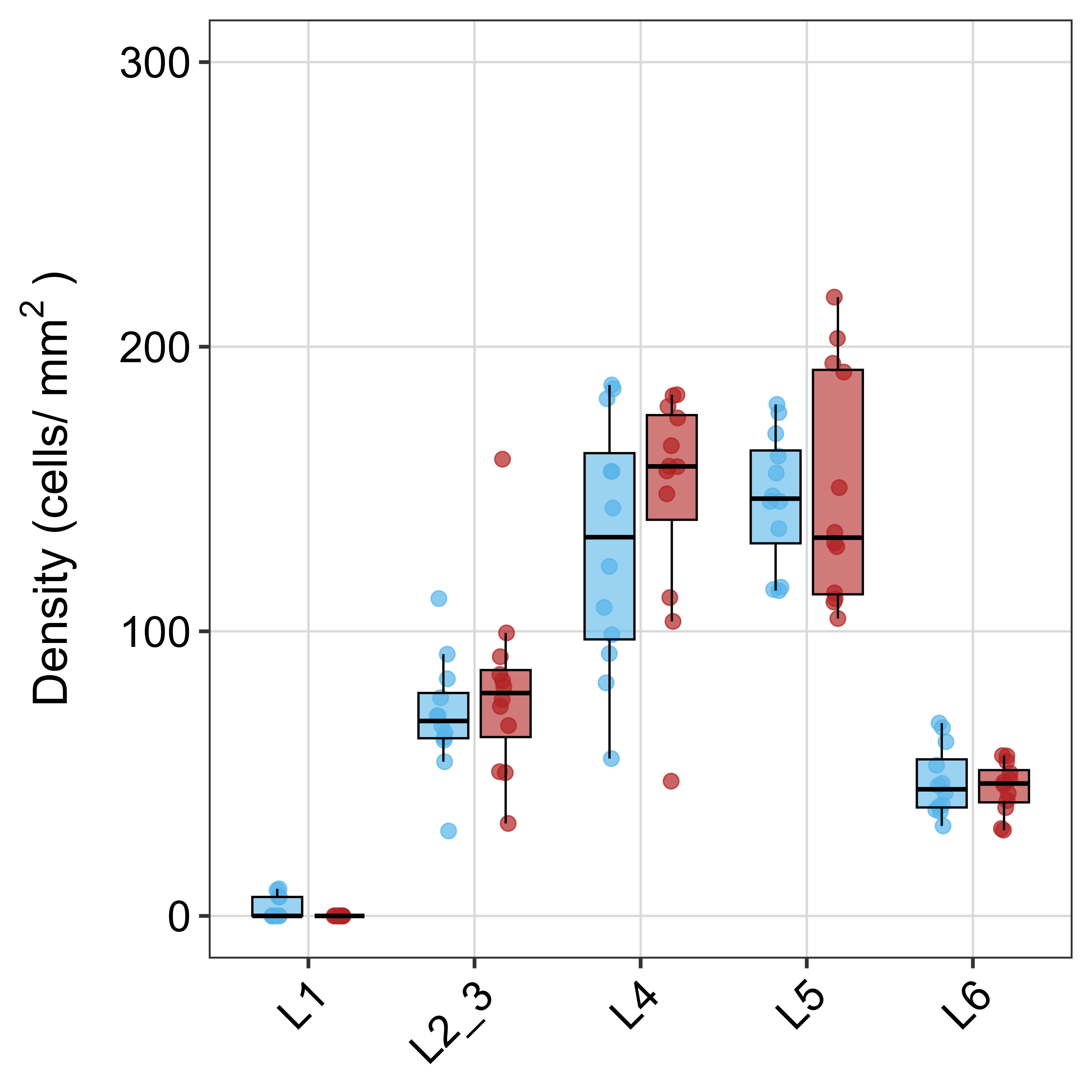

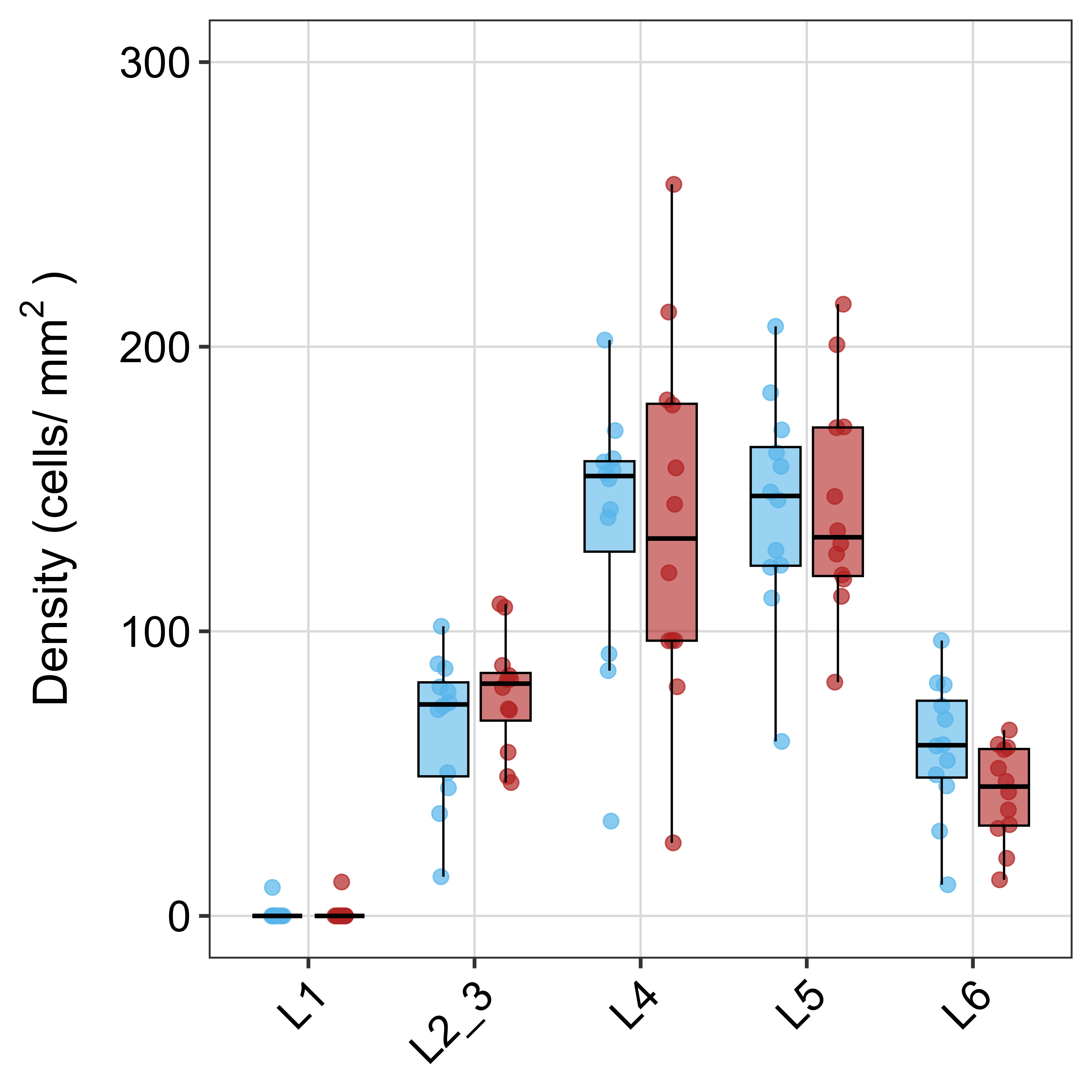

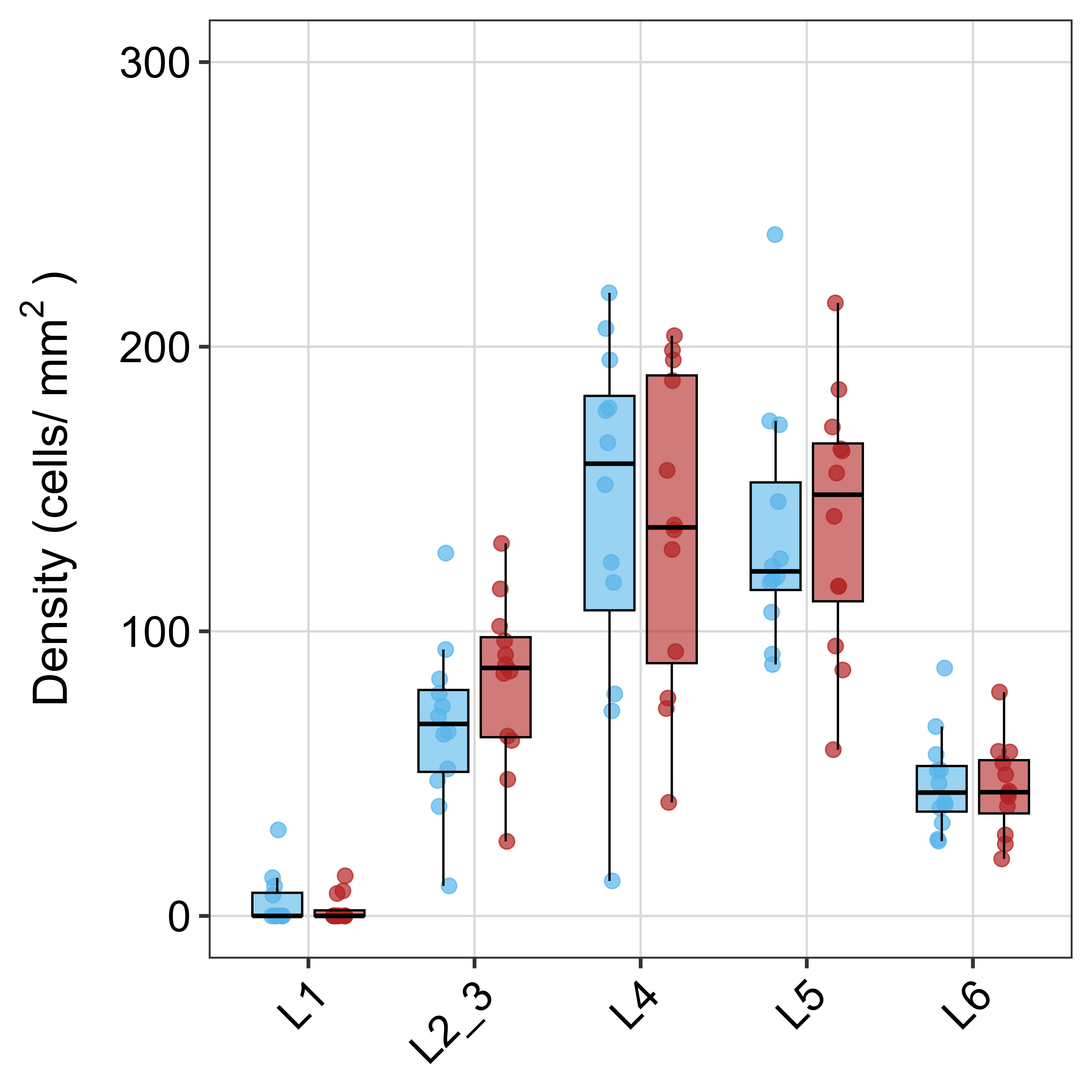


A

B

C

D

E

F

G

H

I

J

K

L

**Figure S3.** Parvalbumin-immunopositive (PV+) interneuron density in the somatosensory cortex (SSC). Overall PV+ cell density in the whole SSC (A), in the upper limb (B), in the mouth (C), and in the nose (D) subregions showed no significant difference between wild type (WT, blue) and *Cntnap2* KO (red) mice. When stratified by sex, we observed no significant difference within females or males between *Cntnap2* KO and WT mice in the overall PV+ cell density (E), upper limb (F), mouth (G) and nose (H) subregions. Layerwise analysis revealed no significant difference in CR+ cell density in the whole SSC (I), upper limb (J), mouth (K) and nose (L) subregions.

| **Whole somatosensory cortex** | | | | |
| --- | --- | --- | --- | --- |
| **Layer** | **p-value** | **df** | **t** | **95% CI** |
| **L1** | 0.8701 | 79.6 | 0.16 | [−19.9, 23.5] |
| **L2/3** | 0.2627 | 79.6 | −1.13 | [−34.0, 9.4] |
| **L4** | 0.5290 | 79.6 | −0.63 | [−28.6, 14.8] |
| **L5** | 0.7703 | 79.6 | −0.29 | [−24.9, 18.5] |
| **L6** | 0.6001 | 79.6 | 0.53 | [−15.9, 27.4] |
| **All layers** | 0.6129 | 21 | 0.51 | [−9.9, 16.5] |
| **Mouth subregion of SSC** | | | | |
| **Layer** | **p-value** | **df** | **t** | **95% CI** |
| **L1** | 0.9909 | 82.8 | −0.01 | [−28.1, 27.7] |
| **L2/3** | 0.4302 | 82.8 | −0.79 | [−39.0, 16.8] |
| **L4** | 0.9809 | 82.8 | 0.02 | [−27.6, 28.2] |
| **L5** | 0.9646 | 82.8 | −0.05 | [−28.5, 27.3] |
| **L6** | 0.2493 | 82.8 | 1.16 | [−11.7, 44.1] |
| **All layers** | 0.9868 | 21 | -0.02 | [−0.21, 0.20] |
| **Nose subregion of SSC** | | | | |
| **Layer** | **p-value** | **df** | **t** | **95% CI** |
| **L1** | 0.8661 | 72.5 | 0.17 | [−27.4, 32.6] |
| **L2/3** | 0.2949 | 72.5 | −1.06 | [−45.9, 14.1] |
| **L4** | 0.6937 | 72.5 | 0.40 | [−24.0, 36.0] |
| **L5** | 0.8016 | 72.5 | −0.25 | [−33.8, 26.2] |
| **L6** | 0.8966 | 72.5 | 0.13 | [−28.0, 32.0] |
| **All layers** | 0.6988 | 21 | 0.39 | [−16.6, 24.4] |
| **Upper limb subregion of SSC** | | | | |
| **Layer** | **p-value** | **df** | **t** | **95% CI** |
| **L1** | 0.8167 | 94.4 | 0.23 | [−20.0, 25.4] |
| **L2/3** | 0.4444 | 94.4 | −0.77 | [−31.5, 13.9] |
| **L4** | 0.1492 | 94.4 | −1.45 | [−39.3, 6.1] |
| **L5** | 0.8344 | 94.4 | −0.21 | [−25.1, 20.3] |
| **L6** | 0.8500 | 94.4 | 0.19 | [−20.5, 24.9] |
| **All layers** | 0.4376 | 21 | 0.79 | [−7.4, 16.6] |

**Table S3.** Summary table of the statistical results from linear mixed model and post hoc comparisons of parvalbumin-immunopositive (PV+) interneuron density in the somatosensory cortex (SSC) between *Cntnap2* KO and wild type mice. No significant difference was found in the overall CR+ cell density or in any examined subregions.

|  | **Males** | **Females** | **Sex×Genotye** | **df** | **t** | **95% CI** |
| --- | --- | --- | --- | --- | --- | --- |
| **Whole SSC** | 0.6932 | 0.5244 | 0.6942 | 21 | -0.41 | [−19.4, 13.0] |
| **Mouth** | 0.9572 | 0.9626 | 0.9530 | 21 | 0.00 | [−0.17, 0.17] |
| **Nose** | 0.4989 | 0.8495 | 0.5234 | 21 | -0.32 | −29.0, 21.2] |
| **Upperlimb** | 0.6932 | 0.5244 | 0.7437 | 21 | 0.79 | [−7.4, 16.6] |

**Table S4.** Summary table of statistical results from linear mixed model and post hoc comparisons of the effect of sex on parvalbumin-immunopositive interneuron density in the somatosensory cortex between *Cntnap2* KO and wild type mice.


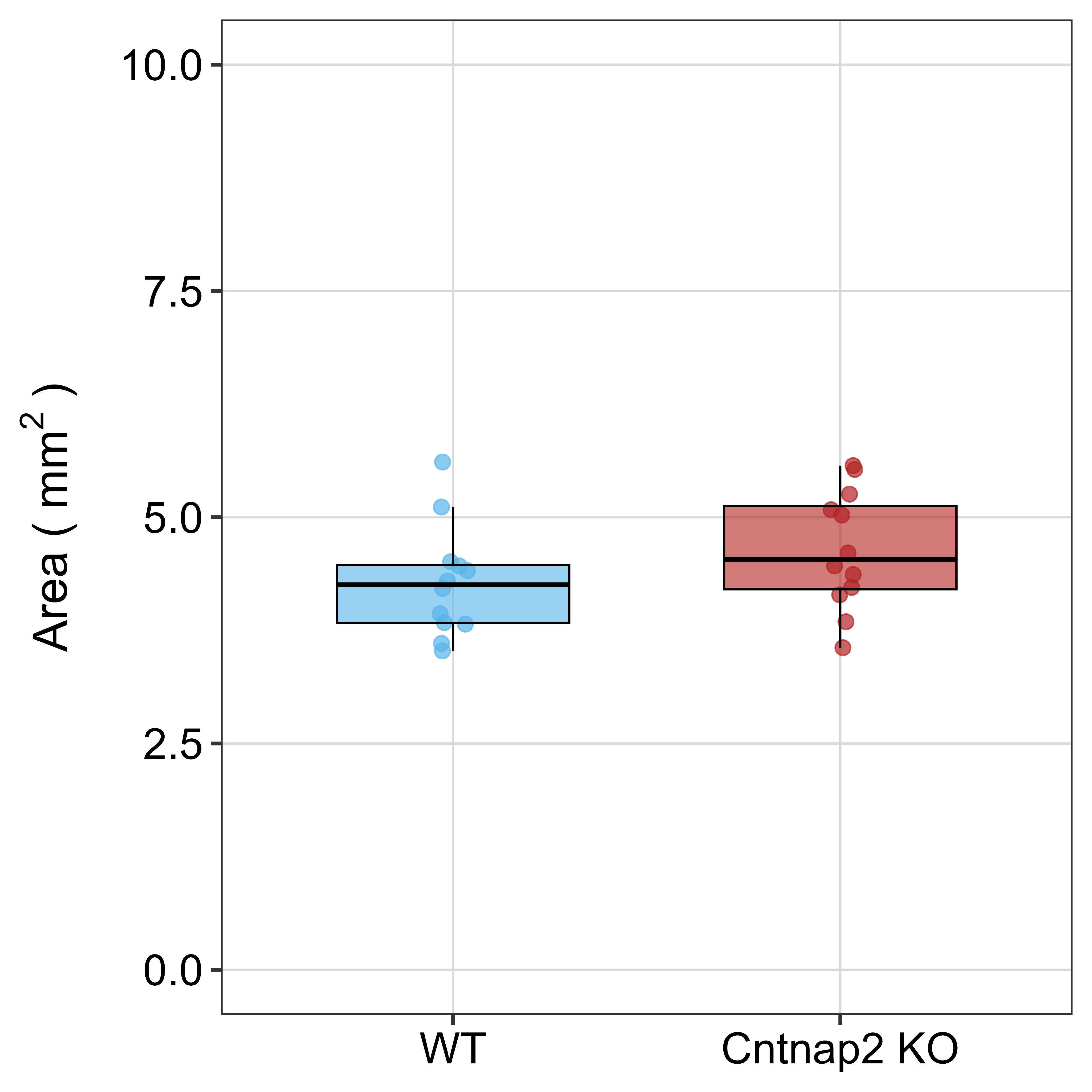

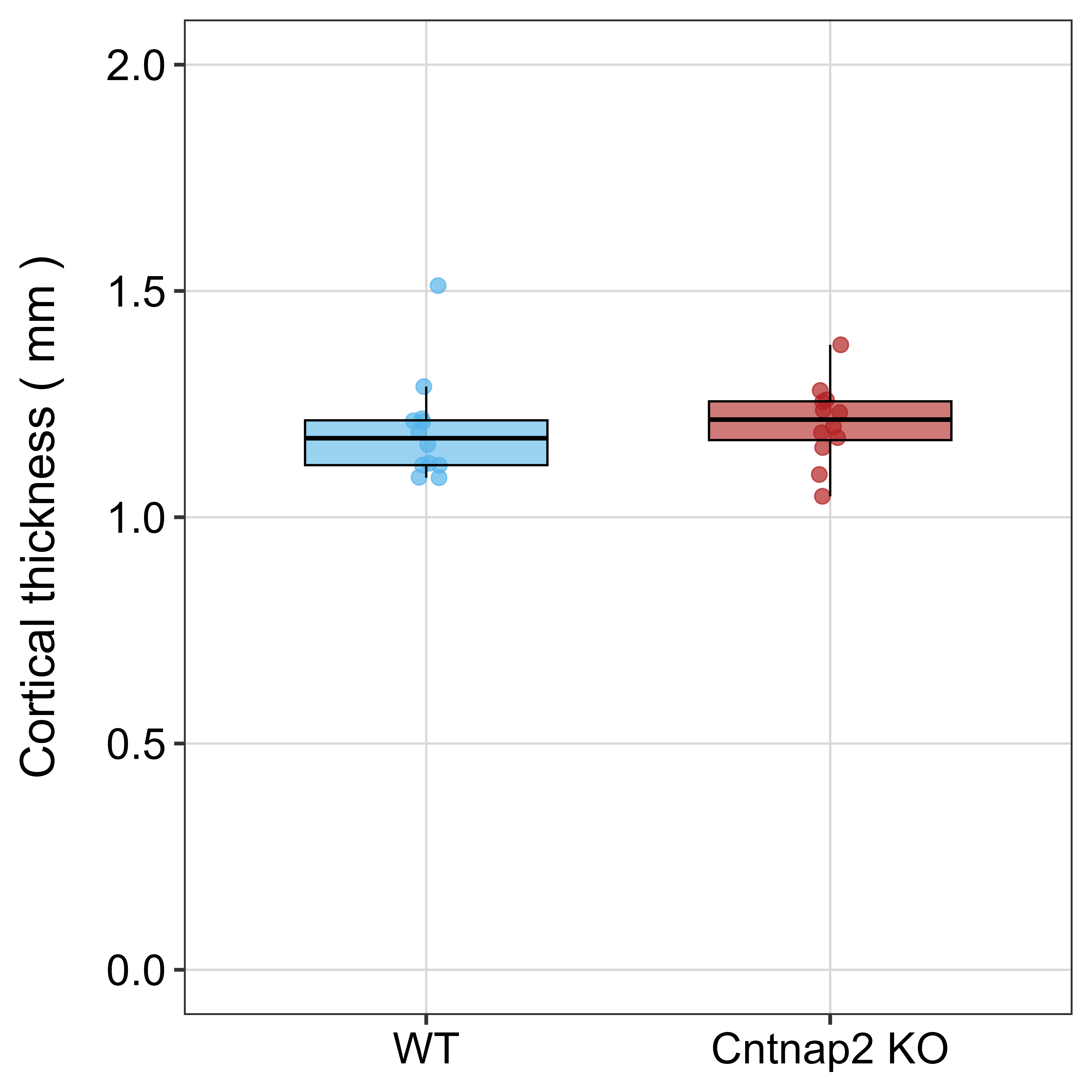


A

B

**Figure S4.** Area of the caudoputamen (CP) and thickness of the somatosensory cortex (SSC) in Cntnap2 KO and WT mice. No significant difference was found in the area of the CP (A, p = 0.1862) or the thickness of the SSC (B, p = 0.7255).

**Social novelty test**

| ***Cntnap2* KO vs. wild-type control male mice** | **p-value** | **df value** | **t value** | **R squared** |
| --- | --- | --- | --- | --- |
| **Time spent with unknown chamber** | 0.8308 | 10 | 0.2193 | 0.004787 |
| **Time spent with known conspecifics** | 0.7290 | 10 | 0.3563 | 0.01254 |
| **Time spent in the middle chamber** | 0.7147 | 10 | 0.3760 | 0.01394 |
| ***Cntnap2* KO vs. wild-type control females** | **p-value** | **df value** | **t value** | **R squared** |
| **Time spent with unknown conspecific** | **0.0083** | 9 | 3.369 | 0.5577 |
| **Time spent with known conspecific** | 0.2287 | 9 | 1.291 | 0.1563 |
| **Time spent in the middle chamber** | **0.0191** | 9 | 2.848 | 0.4741 |

**Table S5.** Summary of the statistical results of the comparison of *Cntnap2* KO and wild type control groups during social novelty test. Unpaired two-tailed Student’s t-test pairwise comparisons were applied to reveal significant differences in the time spent in each chamber. Significant values (p < 0.05) are shown in bold.

| **Time spent with unknown conspecifics vs.  Time spent with known conspecifics** | **p-value** | **df value** | **t value** | **R squared** |
| --- | --- | --- | --- | --- |
| ***Cntnap2* KO females** | **0.0063** | 8 | 3.676 | 0.6281 |
| **Wild-type females** | 0.7091 | 10 | 0.3839 | 0.01453 |
| ***Cntnap2* KO males** | 0.3369 | 10 | 1.009 | 0.09239 |
| **Wild-type males** | 0.2977 | 10 | 1.099 | 0.1077 |
| **Time spent with known conspecifics vs. Time spent in middle chamber** | **p-value** | **df value** | **t value** | **R squared** |
| ***Cntnap2* KO females** | **<0.0001** | 8 | 10.82 | 0.9361 |
| **Wild-type females** | **<0.0001** | 10 | 16.57 | 0.9649 |
| ***Cntnap2* KO males** | **<0.0001** | 10 | 10.67 | 0.9193 |
| **Wild-type males** | **<0.0001** | 10 | 7.922 | 0.8626 |
| **Time spent with unknown conspecifics vs. Time spent in middle chamber** | **p-value** | **df value** | **t value** | **R squared** |
| ***Cntnap2* KO females** | **<0.0001** | 8 | 9.489 | 0.9184 |
| **Wild-type females** | **<0.0001** | 10 | 14.19 | 0.9527 |
| ***Cntnap2* KO males** | **<0.0001** | 10 | 11.62 | 0.9310 |
| **Wild-type males** | **<0.0001** | 10 | 7.476 | 0.8482 |

**Table S6.** Summary of p-values from unpaired two-tailed Student’s t-test pairwise comparisons of time spent in each chamber during the social novelty test. Significant values (p < 0.05) are shown in bold.

Global activity and immobility of *Cntnap2* KO and wild-type mice

| ***Cntnap2* KO vs. wild-type control female mice** | **p-value** | **df value** | **t value** | **R squared** |
| --- | --- | --- | --- | --- |
| **Total traveled distance** | **0.0176** | 9 | 2.900 | 0.4831 |
| **Global activity** | 0.2127 | 9 | 1.341 | 0.1666 |
| **Time spent with immobility** | 0.4050 | 9 | 0.8736 | 0.07817 |
| ***Cntnap2* KO vs. wild-type control male mice** | **p-value** | **df value** | **t value** | **R squared** |
| **Total traveled distance** | 0.0998 | 10 | 1.813 | 0.2475 |
| **Global activity** | 0.3326 | 10 | 1.018 | 0.09394 |
| **Time spent with immobility** | 0.5585 | 10 | 0.6052 | 0.03534 |

**Table S7.** Summary of the statistical results of the comparison of activity and total traveled distance of *Cntnap2* KO and wild-type control groups. Unpaired two-tailed Student’s t-test pairwise comparisons were applied to reveal significant differences in the time spent in each chamber. Significant values (p < 0.05) are shown in bold.

**Direct social behavior tests**

| **Moving away behavior** | **Effect of group (*Cntnap2* KO vs. WT)** | | |
| --- | --- | --- | --- |
|  | **Df values** | **F value** | **p value** |
| Frequency | 1, 22 | 19.94 | **0.00021** |
| Duration | 1, 22 | 8.99 | **0.00662** |
| Latency | 1, 22 | 6.394 | **0.01913** |
| **Moving away behavior** | **Effect of sex (male vs. female)** | | |
|  | **Df values** | **F value** | **p value** |
| Frequency | 1, 21 | 2.9944 | 0.0982235 |
| Duration | 1, 21 | 0.3198 | 0.577723 |
| Latency | 1, 21 | 2.0892 | 0.16310 |
| **Moving away behavior** | **Effect of group x sex interaction** | |  |
|  | **Df values** | **F value** | **p value** |
| Frequency | 1, 20 | 0.322 | 0.5767 |
| Duration | 1, 20 | 0.1139 | 0.7393 |
| Latency | 1, 20 | 3.0155 | 0.09785 |
| **Passive social behavior** | **Effect of group (*Cntnap2* KO vs. WT)** | | |
|  | **Df values** | **F value** | **p value** |
| Frequency | 1, 22 | 0.215 | 0.6474 |
| Duration | 1, 22 | 1.223 | 0.2807 |
| Latency | 1, 22 | 1.562 | 0.2245 |
| **Approaching** | **Effect of group (*Cntnap2* KO vs. WT)** | | |
|  | **Df values** | **F value** | **p value** |
| Frequency | 1, 22 | 0.813 | 0.377 |
| Duration | 1, 22 | 0.275 | 0.6052 |
| Latency | 1, 22 | 0.1959 | 0.6624 |

**Table S8.** P-values for the effect of group, sex, and their interaction on behavior elements. Linear regression model was applied. Significant values (p < 0.05) are shown in bold.

| Correlation analysis between CR cell density in the caudoputamen and moving away behavior | | | | |
| --- | --- | --- | --- | --- |
| **Frequency** | **Df values** | **F value** | **Adjusted R^2^ value** | **p value** |
| **Full model (linear model)** | 3, 20 | 14.01 | 0.6293 | **<0.001** |
| **Effect of group x density interaction** | 1, 20 | 6.081 | - | **0.02283** |
| **Effect of CR density within group** | **Slope (β)** | **SE value** | **t value** | **p value** |
| *Cntnap2* KO mice | 4.76 | 1.300 | 3.648 | **0.0016** |
| Wild-type mice | 0.74 | 0.977 | 0.757 | 0.4577 |
| **Duration** | **Df values** | **F value** | **Adjusted R^2^ value** | **p value** |
| **Full model (linear model)** | 3, 20 | 11.01 | 0.5663 | **<0.001** |
| **Effect of group x density interaction** | 1, 20 | 9.0633 | - | **0.00691** |
| **Effect of CR density within group** | **Slope (β)** | **SE value** | **t value** | **p value** |
| *Cntnap2* KO mice | 6.019 | 1.44 | 4.166 | **0.0005** |
| Wild-type mice | 0.585 | 1.08 | 0.540 | 0.5951 |
| **Latency** | **Df values** | **F value** | **Adjusted R^2^ value** | **p value** |
| **Full model (linear model)** | 3, 20 | 2.901 | 0.1987 | 0.06026 |
| **Effect of group x density interaction** | 1, 20 | 0.3876 | - | 0.5406 |
| **Effect of CR density within group** | **Slope (β)** | **SE value** | **t value** | **p value** |
| *Cntnap2* KO mice | -0.985 | 3.10 | -0.318 | 0.7540 |
| Wild-type mice | -3.395 | 2.32 | -1.463 | 0.1591 |

**Table S9.** P-values for the effect of calretinin-immunopositive (CR+) interneuron densities in the caudoputamen on moving away behavior. Linear regression model was applied. Significant values (p < 0.05) are shown in bold.

| Correlation analysis between CR cell density in the somatosensory cortex and moving away behavior | | | | |
| --- | --- | --- | --- | --- |
| **Frequency** | **Df values** | **F value** | **Adjusted R^2^ value** | **p value** |
| **Full model (linear model)** | 3, 20 | 7.947 | 0.4754 | 0.001105 |
| **Effect of group x density interaction** | 1, 20 | 3.7516 | - | 0.06701 |
| **Duration** | **Df values** | **F value** | **Adjusted R^2^ value** | **p value** |
| **Full model (linear model)** | 3, 20 | 4.703 | 0.3257 | 0.01213 |
| **Effect of group x density interaction** | 1, 20 | 3.4626 | - | 0.07754 |
| **Latency** | **Df values** | **F value** | **Adjusted R^2^ value** | **p value** |
| **Full model (linear model)** | 3, 20 | 2.082 | 0.1237 | 0.1347 |
| **Effect of group x density interaction** | 1, 20 | 0.2964 | - | 0.5922 |

**Table S10.** P-values for the effect of calretinin-immunopositive (CR+) interneuron densities in the somatosensory cortex on moving away behavior. Linear regression model was applied. Significant values (p < 0.05) are shown in bold.
